# Model‐Guided Systematic Metabolic Engineering for Enhanced Spinosad Biosynthesis in *Saccharopolyspora spinosa* NHF132

**DOI:** 10.1002/advs.202511411

**Published:** 2025-09-26

**Authors:** Shuliu Wang, Yuxin Liu, Qian Zhang, Yue Jiang, Xiaoqian Zeng, Chengyu Zhang, Jiagao Cheng, Weishan Wang, Lixin Zhang, Gao‐Yi Tan

**Affiliations:** ^1^ State Key Laboratory of Bioreactor Engineering (SKLBE) and School of Biotechnology East China University of Science and Technology (ECUST) Shanghai 200237 China; ^2^ Shanghai Key Laboratory of Chemical Biology School of Pharmacy East China University of Science and Technology Shanghai 200237 China; ^3^ State Key Laboratory of Microbial Diversity and Innovative Utilization Institute of Microbiology Chinese Academy of Sciences (CAS) Beijing 100101 China

**Keywords:** genome‐scale metabolic model, *Saccharopolyspora spinosa*, secondary metabolite, spinosad, systems metabolic engineering

## Abstract

Spinosad (a mixture of spinosyns A and D) is a macrocyclic lactone green bioinsecticide produced by *Saccharopolyspora spinosa*. It is known for its high efficiency, low toxicity, and broad‐spectrum activity. Although numerous strategies have been employed to enhance spinosad production, intricate regulation of secondary metabolism and inefficient genetic manipulation impede systematic and comprehensive metabolic engineering in this spinosad‐producing strain. In this study, a genome‐scale metabolic model (GEM) for *Sa. spinosa* NHF132 is developed to dissect the intricate secondary metabolic pathways of spinosad biosynthesis, analyzing interactions among precursors, key enzymes, and competing or bypass pathways. Guided by the model, the impact of rhamnose precursor overexpression, gene cluster amplification, short‐chain acyl‐CoA enhancement, and chassis optimization on spinosad production is systematically evaluated. By integrating these metabolic engineering strategies, engineered strain NHF132‐BAC‐*SP43*‐NCM achieved a spinosad titer of 1816.8 mg L^−1^, a 553.3% increase over the starting strain, with substantial improvements in yield and product proportion. The model‐driven framework for metabolic engineering of complex secondary metabolites in actinomycetes substantially increased spinosad production and offered valuable insights for other complex natural products.

## Graphical abstract

A genome‐scale model guided the systematic metabolic engineering of *Saccharopolyspora spinosa*, enabling a 5.5‐fold boost in the green bioinsecticide spinosad to 1.8 g L^−1^ and establishing an actinomycete framework for complex natural products overproduction.

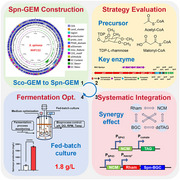

## 1 Introduction

Spinosad, a macrolide secondary metabolite mixture produced by the actinomycete *Saccharopolyspora spinosa*, primarily comprises two active components: spinosyn A (accounting for ≈85% of spinosad) and spinosyn D (≈15%).^[^ ^1^ ^]^ The main structural distinction between spinosyn D and A is a methyl substituent at the six‐position of the polyketide backbone.^[^ ^2^ ^]^ Spinosad is an eco‐friendly bioinsecticide applied extensively in agriculture to manage Lepidoptera, Diptera, and Thysanoptera pests.^[^ ^3^, ^4^ ^]^ Its structural diversity confers unique bioactivities and application potential. Spinosyn A and its derivative LM‐2I can inhibit the proliferation and growth of breast cancer cells,^[^ ^5^ ^]^ while spinosyn J and L can be chemically modified to produce the biodegradable pesticide spinetoram.^[^ ^6^ ^]^

Structurally, spinosyns comprise a tetracyclic aglycone, a thymidine diphosphate (TDP)‐L‐rhamnose, and a TDP‐D‐forosamine. Their biosynthesis is governed by a ≈74 kb multi‐module biosynthetic gene cluster (BGC) containing 19 open reading frames (*spnA*−*spnS*).^[^ ^2^ ^]^ Notably, four rhamnose biosynthesis genes (*gtt*, *gdh*, *epi*, and *kre*) are located outside the spinosyn gene cluster. Subsequent studies elucidated the spinosad biosynthetic pathway.^[^ ^1^, ^7^ ^]^ Spinosad is a typical polyketide that predominantly utilizes short‐chain acyl‐coenzyme A (acyl‐CoA) molecules as starter and extender units. There is a close link between spinosad biosynthesis and primary metabolism in *Sa. spinosa*.^[^ ^8^ ^]^ Primary metabolism supplies abundant precursors and energy for efficient spinosad biosynthesis, but there exists some competition between the biosynthesis of secondary metabolites and the basic growth needs of the strain.^[^ ^9^ ^]^

To enhance the spinosad yield, many strategies have been employed including 1) overexpression of biosynthetic genes, including polyketide synthase (PKS), the rhamnose BGC, and forosamine biosynthesis‐related genes^[^ ^10^, ^11^, ^12^, ^13^ ^]^; 2) enhancement of precursor supply, such as acetyl‐CoA and malonyl‐CoA^[^ ^14^, ^15^ ^]^; 3) multi‐omics analysis and metabolic network optimization, including transcriptomics and metabolomics^[^ ^16^, ^17^, ^18^ ^]^; 4) heterologous biosynthesis^[^ ^19^, ^20^, ^21^, ^22^ ^]^; 5) medium optimization and fermentation process improvement^[^ ^23^, ^24^ ^]^; 6) mutagenesis breeding^[^ ^25^, ^26^ ^]^; and 7) combinatorial strategies.^[^ ^27^, ^28^ ^]^ However, the genetic intractability of *Sa. spinosa* poses a major barrier to the implementation of diverse metabolic engineering approaches; there are few examples of systematic metabolic engineering in *Sa. spinosa*. Furthermore, owing to the challenges of genetic manipulation, the intricacy of secondary metabolism, and the complexity of the yet‐unknown regulatory networks in actinomycetes,^[^ ^29^ ^]^ these single‐factor strategies typically achieve only modest improvement.

The present study developed an actinomycete genome‐scale metabolic model (Spn‐GEM; https://github.com/Shuliu97/Spn_GEM) to analyze the secondary metabolic process of spinosyns production in *Sa. spinosa* NHF132. This model clarified interactions among precursors, key enzymes, and competing/parallel pathways, and identified several potential targets. Subsequently, a range of metabolic engineering strategies including rhamnose precursor overexpression, complete BGC overexpression, short‐chain acyl‐CoA enhancement, and chassis engineering were evaluated for their impact on spinosyns production. Based on the Spn‐GEM, effective strategies were rationally combined to achieve additive and synergistic effects. Notably, combining BGC amplification and the non‐carboxylative malonyl‐CoA (NCM) pathway had a synergistic effect, increasing production 4.1‐fold to 1405.4 mg L^−1^. Following further fermentation optimization, spinosad production reached 1816.8 mg L^−1^, a 553.3% increase over the original strain. These results demonstrate the advantages of model‐guided combinatorial metabolic engineering for efficient spinosad biosynthesis.

## 2 Results and Discussion

### 2.1 Spn‐GEM Construction and Analysis of Spinosad Biosynthesis

Actinomycetes possess a complex metabolic network and are an important source of natural products (NPs) with industrial and medicinal potential.^[^ ^30^ ^]^ Production of secondary metabolites is closely associated with primary metabolism.^[^ ^8^ ^]^ Based on genome annotation, GEMs provide a systemic view of metabolism. They integrate strain‐specific genes, proteins, and pathway knowledge, and thereby act as a metabolic target prediction tool. GEMs are widely used to accelerate cell factory construction in metabolic engineering. For example, a GEM was used to improve FK506 production 1.47‐fold in *Streptomyces tsukubaensis*.^[^ ^31^ ^]^

**Figure 1:**
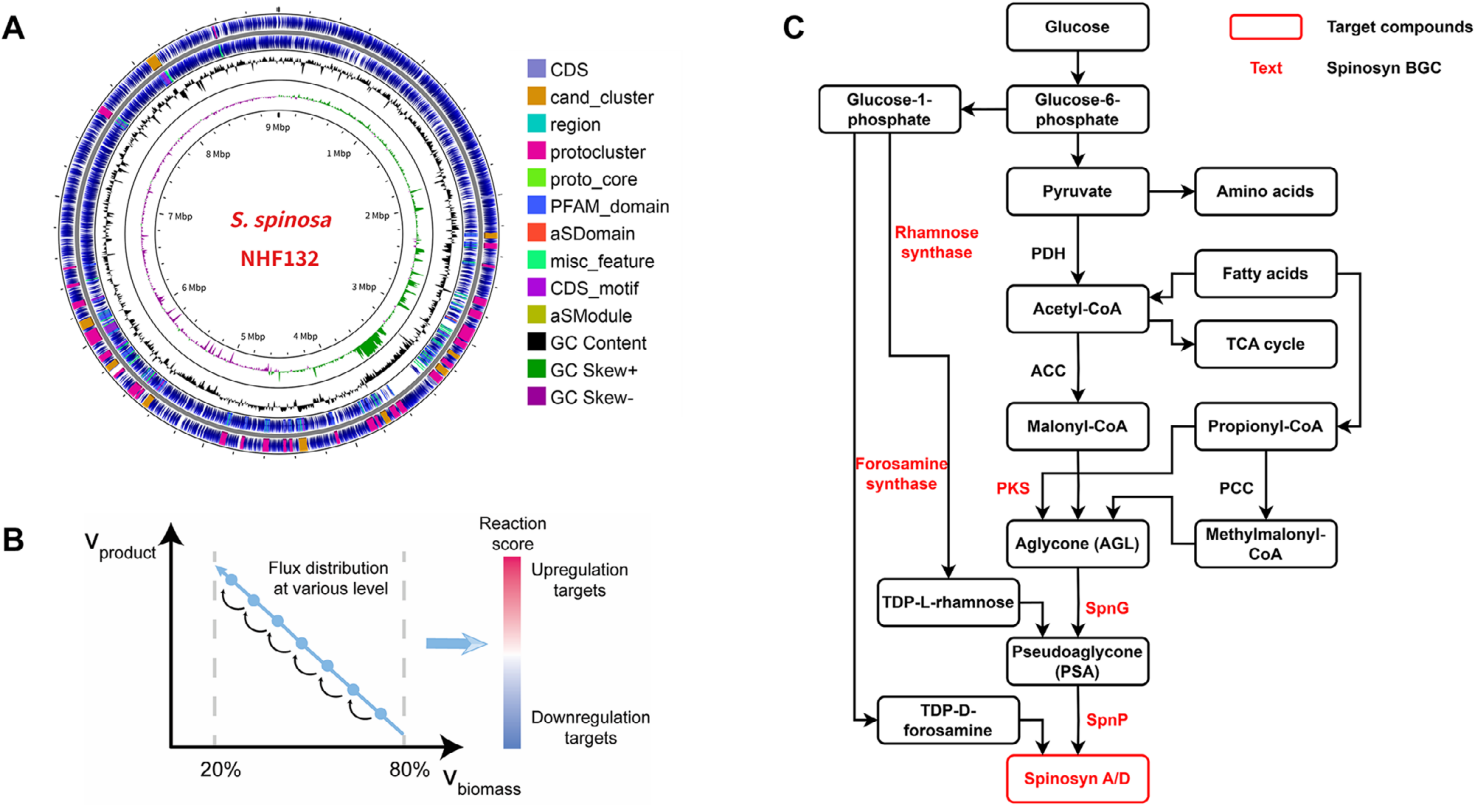
Genome annotation and spinosad biosynthetic pathway modeling. A) Genome annotated with antiSMASH,^[^ ^35^ ^]^ visualized with Proksee.^[^ ^36^ ^]^ CDS, coding sequence. cand_cluster, candidate cluster. aSDomain (aSModule), antiSMASH secondary‐metabolite domain (module). misc_feature, miscellaneous feature. B) Target prediction by FSEOF for spinosad overproduction (growth rate fixed at 20–80% of the maximum). C) Biosynthesis pathway from carbon sources to spinosad production. Spinosyn A/D are the target compounds, with red text indicating the parts contained in the BGC. TCA, tricarboxylic acid. PDH, pyruvate dehydrogenase. ACC, acetyl‐CoA carboxylase. PCC, propionyl‐CoA carboxylase.

The non‐model actinomycete *Sa. spinosa* possesses numerous metabolites and unique physiological traits, resulting in a highly intricate metabolic network (**Figure** **1**A). By integrating the spinosad biosynthetic module (including the synthesis of the aglycone, forosamine, pseudoaglycone, and spinosyn A) into the classic Sco‐GEM of *S. coelicolor*, we constructed a preliminary Spn‐GEM for *Sa. spinosa* NHF132.^[^ ^32^ ^]^ Modified flux scanning based on enforced objective flux (FSEOF) was used to identify targets.^[^ ^33^, ^34^ ^]^ By simulating changes in physiological conditions under different suboptimal growth rates and maximizing spinosad yield, we ran multiple simulations using glucose as the carbon source with a theoretical maximum biomass from 20% to 80% as stepwise constraints (Figure 1B). After each simulation, parsimonius flux balance analysis (pFBA) was performed to minimize total flux and output the final flux distribution (Table S7, Supporting Information). Based on the flux distribution, reaction scores and gene scores were calculated using the previously established strategy (Table S8, Supporting Information). The score for reaction A is defined as the ratio of its flux under a biomass‐production regime (growth rate fixed at 20–80% of the maximum) to the flux observed during pure biomass maximization. Thus, a score exceeding 1 signifies that flux surpasses control conditions (0% production and 100% growth), implying that the reaction should be upregulated—and vice versa. Our prediction contained 36 upregulation targets and 20 downregulation targets (Table S9, Supporting Information), among which we rationally chose seven (Table S9, Supporting Information, targets 1−4, 6, 9, 56) for in vivo estimation.

Based on Spn‐GEM, the potential principal bottlenecks for spinosad production in NHF132 were 1) expression of the spinosad biosynthetic module (Table S9, Supporting Information, targets 1−4); 2) rhamnose biosynthesis (Table S9, Supporting Information, targets 6, 9, 56); and 3) short‐chain acyl‐CoA supply (Table S9, Supporting Information, targets 26, 32, 39, 44, 45, 49, 50, 52). Meanwhile, based on prior studies, we outlined the biosynthesis pathway from carbon sources to spinosad production, marking related bypaths such as fatty acids and amino acids, and highlighting the conversion relationship between key acyl‐CoA precursors (Figure 1C).

**Figure 2:**
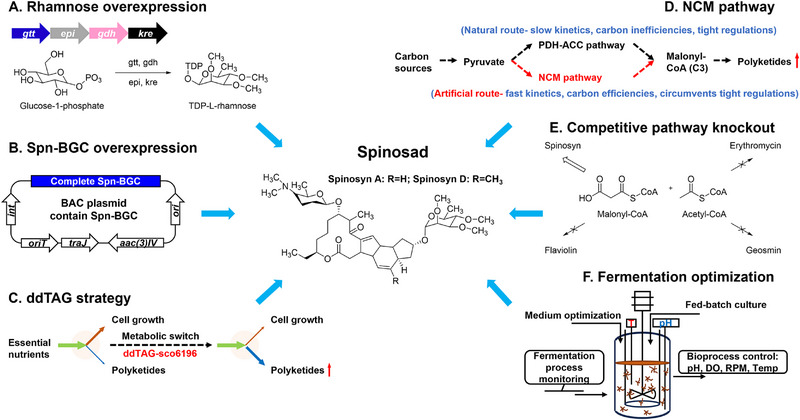
Diagram summarizing the strategies utilized in the present study. A) Overexpression of *gtt*, *epi*, *gdh*, *and kre* genes to enhance the supply of the precursor TDP‐L‐rhamnose. B) Overexpression of the complete spinosad BGC via bacterial artificial chromosome (BAC) integration. C) Introduction of the ddTAG strategy to adjust metabolic flux. D) Introduction of the artificial NCM pathway to improve malonyl‐CoA flux. E) Knockout of competitive BGCs. F) Optimization of the fermentation process via bioreactor monitoring of pH, dissolved oxygen (DO), and temperature.

Based on Spn‐GEM model prediction with multidimensional metabolic engineering principles, we systematically optimized spinosad production through combinatorial metabolic engineering strategies, including rhamnose precursor overexpression, cluster amplification, short‐chain acyl‐CoA enhancement, and chassis engineering (**Figure** **2**). Among them, overexpression of the rhamnose gene cluster and the complete spinosad BGC aimed to enhance the expression levels of sugar donors and the PKS skeleton (Figure 2A,B). The dynamic degradation of triacylglycerols (ddTAG) strategy aimed to balance strain growth and production, redirecting metabolic flux from cell growth toward secondary metabolite synthesis during the late‐stage growth phase (Figure 2C). The artificial NCM pathway could save carbon sources and enhance malonyl‐CoA flux by directly catalyzing the conversion of pyruvate to malonyl‐CoA with fast kinetics and bypassing tight regulation (Figure 2D). Meanwhile, we knocked out competitive BGCs to channel short‐chain acyl‐CoA supply toward spinosad production (Figure 2E). Finally, we employed fermentation optimization to elevate the spinosad yield further. This involved refining the medium composition and process parameters (Figure 2F).

### 2.2 Overexpression of the Rhamnose Biosynthetic Pathway in *Sa. spinosa*

**Figure 3:**
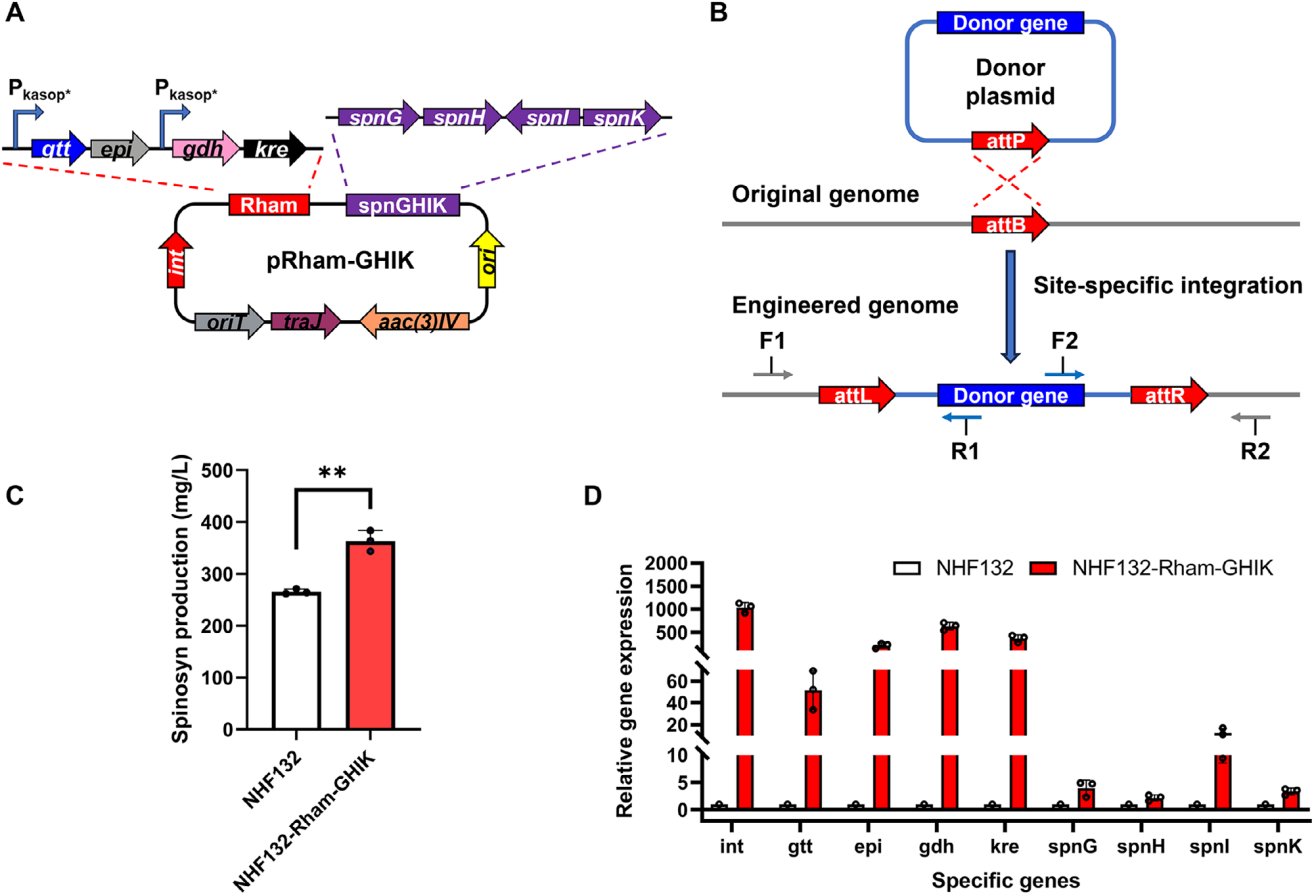
Overexpression of the rhamnose biosynthetic module for spinosad overproduction in *Sa. spinosa* NHF132. A) Rhamnose overexpression plasmid pRham‐GHIK. *int*, integrase gene. *oriT*, RK2 conjugative cis‐acting element. *traJ*, oriT‐recognizing protein. *aac(3)IV*, apramycin resistance gene. *ori*, *colE1* replication origin. B) Schematic diagram of donor plasmid integration into the *Sa. spinosa* chromosome via site‐specific recombination at the *attP/attB* sites. *attP*, attachment site on the donor plasmid. *attB*, chromosomal attachment site. *attL* and *attR*, left and right attachment sites generated after site‐specific recombination between *attP* and *attB*, respectively. F1/R2, genome‐specific primers; F2/R1, gene‐specific primers (sequences in Table S3, Supporting Information). C) Spinosad (spinosyn A/D) production in wild‐type strain NHF132 and engineered strain NHF132‐Rham‐GHIK overexpressing the rhamnose biosynthetic module. D) qPCR analysis of rhamnose‐related genes in NHF132 and NHF132‐Rham‐GHIK in fermentation broth on day 6. Bar graphs with error bars show means ± standard deviation (SD) of three independent experiments (*n* = 3). Statistical significance was assessed by Student's *t*‐test (C) or one‐way ANOVA (D). **p < 0.01.

Rhamnose is a key component of spinosad and expression of its biosynthetic genes is directly linked to spinosad yield. Introducing additional copies of *gtt and epi* under the control of strong promoters such as ermE* can enhance rhamnose availability, thereby boosting spinosad production.^[^ ^10^ ^]^ Rhamnose is also an integral component of the cell wall and is essential for maintaining cellular integrity and morphology. Its deficiency can lead to hyphal fragmentation and impaired growth.^[^ ^37^ ^]^ The Spn‐GEM also demonstrated the need for upregulated expression of rhamnose synthesis‐related genes dTDP‐glucose‐4,6‐dehydratase, dTDP‐4‐dehydrorhamnose‐3,5‐epimerase, and dTDP‐4‐dehydrorhamnose reductase. The rhamnose gene cluster, including *gtt, epi, gdh, and kre*, was overexpressed under the control of the strong promoter kasop*. To channel more rhamnose toward spinosad synthesis, we also cloned the *spnG, H, I, and K* genes, which are directly involved in spinosad production. These genetic elements were integrated into the conjugative transfer vector pSET152 via homologous recombination, resulting in rhamnose overexpression plasmid pRham‐GHIK (**Figure** **3**A).

Through biparental conjugative transfer, we successfully obtained the rhamnose‐overexpressing strain NHF132‐Rham‐GHIK (Figure 3B). Fermentation experiments further demonstrated a enhancement in spinosad titer, which increased by 36.9% from 265.8 to 363.7 mg L^−1^ (Figure 3C). Quantitative PCR (qPCR) analysis of fermentation products revealed a 51.5‐ to 629.7‐fold increase in the expression levels of rhamnose synthesis‐related genes *gtt*, *epi*, *gdh*, and *kre* under the control of kasop* (Figure 3D). Under the control of the natural promoter, expression levels of *spnG, H, I*, and *K* increased by 2.2‐ to 12.5‐fold. These results indicate that overexpression of rhamnose and its associated modification genes benefits spinosad biosynthesis. However, spinosad yields were not increased by as much as reported in previous studies, suggesting that the rhamnose content in NHF132 may not be the key factor limiting spinosad production.^[^ ^10^, ^38^ ^]^

### 2.3 Complete Spinosad BGC Doubling in *Sa. spinosa*

Amplifying BGCs is a relatively simple and effective strategy to enhance NP yields.^[^ ^39^ ^]^ Previous studies demonstrated that increasing the BGC copy number can markedly improve spinosad production in a heterologous *Streptomyces* chassis. A 224‐fold increase in spinosad yield was achieved by increasing the BGC to five copies using the ZouA system in *S. coelicolor*, raising the titer from 5.6 µg L^−1^ in the parent strain to 1253.9 µg L^−1^.^[^ ^22^ ^]^ A 124% increase in spinosad yield was achieved in *Sa. spinosa* by overexpressing the complete 74 kb spinosyn gene cluster.^[^ ^40^ ^]^ BGC amplification may upregulate the transcription of related genes, thereby enhancing spinosad biosynthesis.

**Figure 4:**
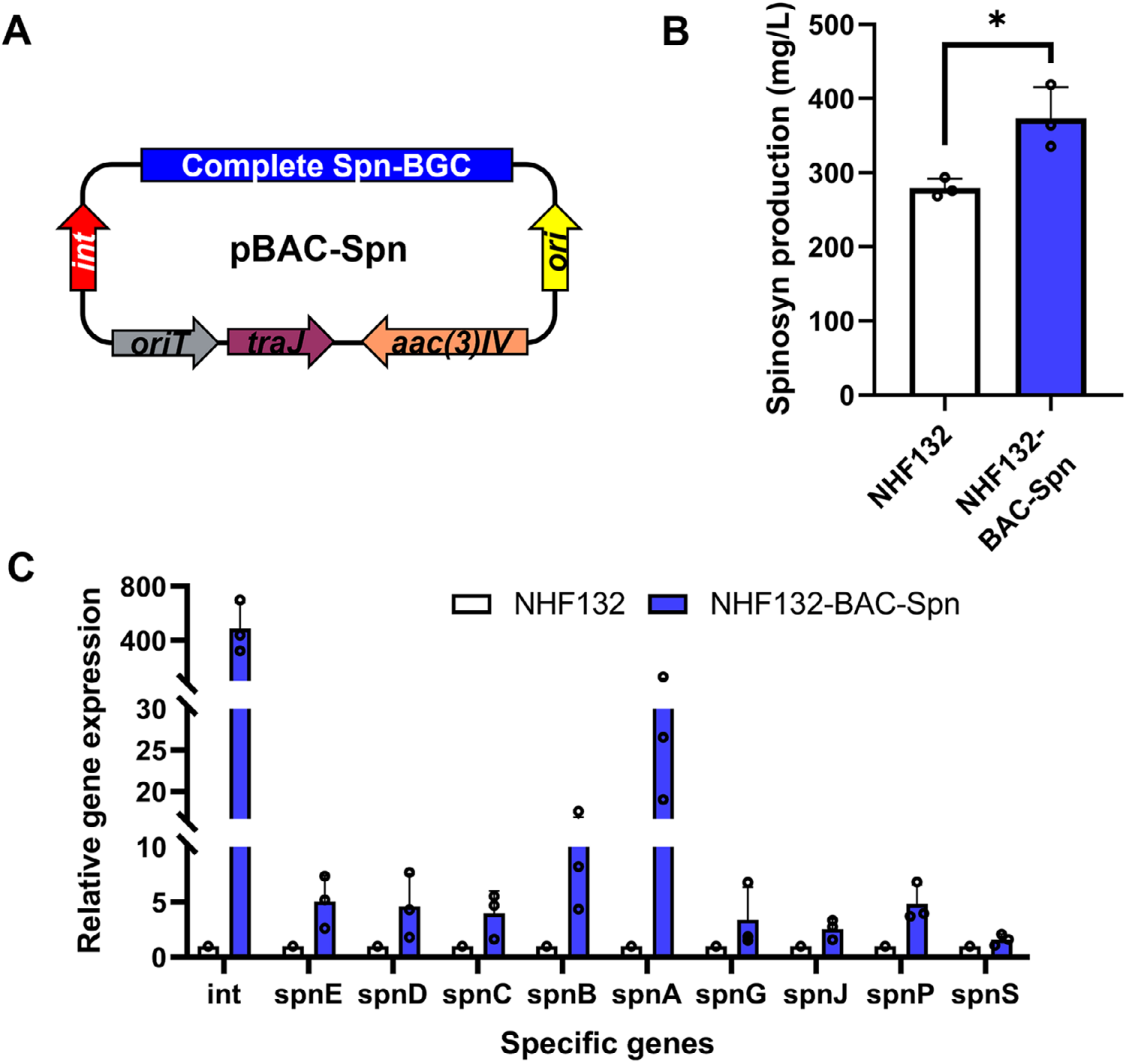
Overexpression of the complete spinosad BGC for spinosad overproduction in *Sa. spinosa* NHF132. A) BGC overexpression plasmid pBAC‐Spn. The complete Spn‐BGC containing 19 genes (*spnA*−*spnS*). B) Spinosad production in NHF132 and engineered strain NHF132‐BAC‐Spn overexpressing the complete spinosad BGC. C) qPCR analysis of specific PKS‐related genes in NHF132 and NHF132‐BAC‐Spn in fermentation broth on day 6. Bar graphs with error bars show means ± SD (*n* = 3). Statistical significance was assessed by Student's *t*‐test (B) or one‐way ANOVA (C). *p < 0.05.

In this study, building upon our previous work, we obtained the complete spinosad BGC and constructed the 81 kb BAC‐based expression vector pBAC‐Spn (**Figure** **4**A).^[^ ^41^ ^]^ Through biparental conjugative transfer, we successfully obtained the BGC‐duplicated strain NHF132‐BAC‐Spn. By duplicating the complete 74 kb spn‐BGC we achieved a 33.6% enhancement in spinosad titer from 279.2 to 373.0 mg L^−1^ (Figure 4B). qPCR analysis also revealed a 1.6‐ to 58.5‐fold increase in the expression levels of genes contained in the BGC in the BGC‐duplicated strain (Figure 4C). These results demonstrated that doubling the BGC is an effective strategy for enhancing spinosad production. However, the modest increase in spinosad yield suggests that Spn‐BGC overexpression alone remains insufficient to achieve a marked titer increase in NHF132.

### 2.4 Introduction of an Additional ddTAG Pathway to Switch Metabolic Flow in *Sa. spinosa*

In actinomycetes, phospholipids (PLs) and TAGs are the main components of cell lipids.^[^ ^42^, ^43^ ^]^ Both accumulate during primary metabolism,^[^ ^44^ ^]^ but when bacteria enter the stable phase and secondary metabolism is triggered,^[^ ^45^, ^46^ ^]^ although the content of phospholipids remains relatively constant, the level of triacylglycerol quickly declines as it is used in the synthesis of secondary metabolites.^[^ ^9^ ^]^

**Figure 5:**
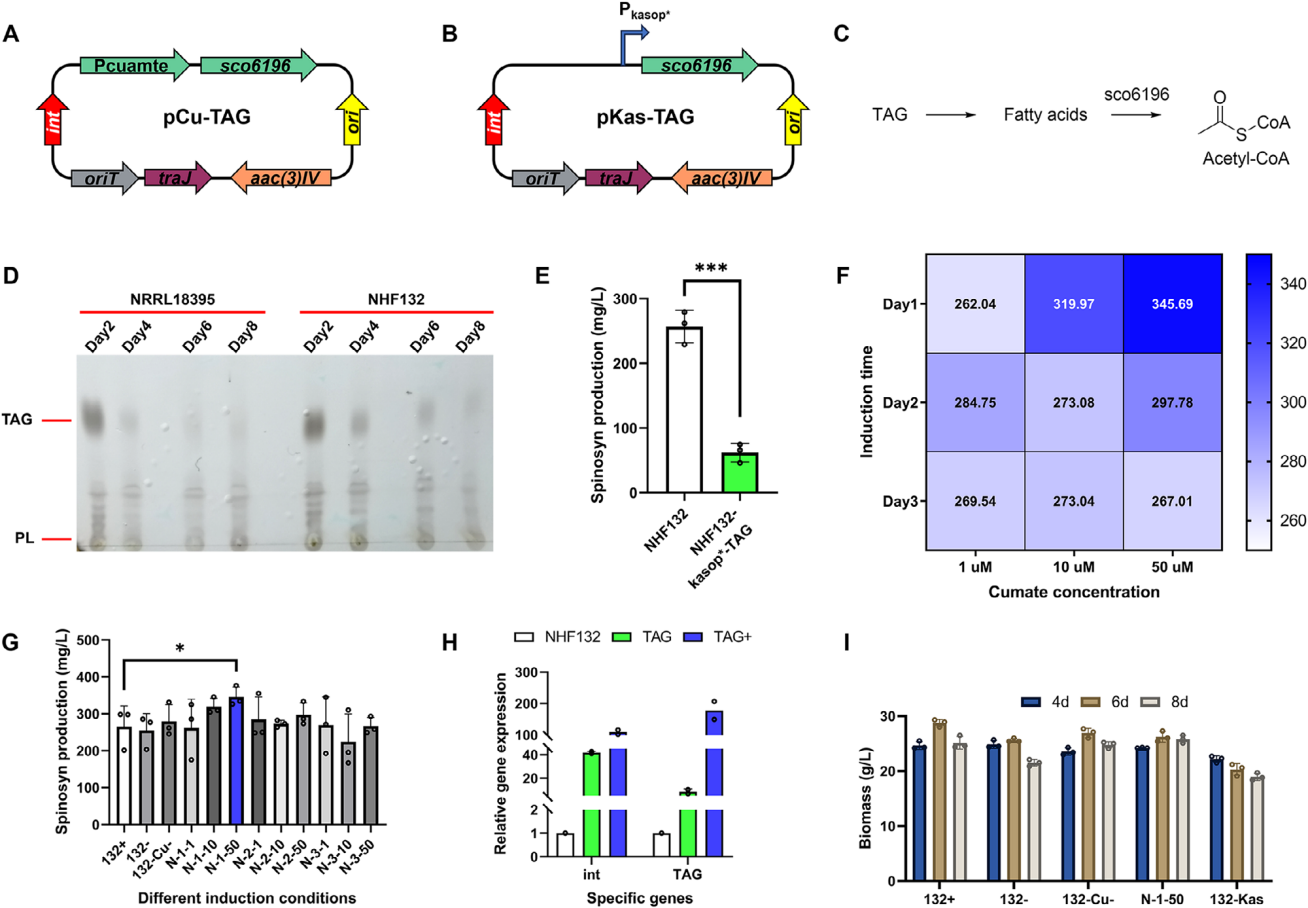
The ddTAG strategy for spinosad overproduction in *Sa. spinosa* NHF132. A) Metabolic flow switching plasmid pCu‐TAG. Pcumate, cumate‐inducible promoter. B) Metabolic flow switching plasmid pKas‐TAG. C) Schematic diagram of the ddTAG strategy. D) TLC analysis of intracellular TAG levels in *Sa. spinosa* NRRL 18395 and NHF132 during fermentation. E) Spinosad (spinosyn A/D) production in wild‐type strain NHF132 and engineered strain NHF132‐kasop*‐TAG. F) Determination of the optimal conditions for spinosad production in *Sa. spinosa*. The colored bar on the right indicates the spinosad titer (the darker the color the higher the yield). G) Spinosad (spinosyn A/D) production in wild‐type strain NHF132 and engineered strain NHF132‐*cumate*‐TAG under different induction conditions. H) qPCR analysis of TAG‐related gene *sco6196* in NHF132 and NHF132‐*cumate*‐TAG in fermentation broth on day 6. I) Biomass measurements of NHF132, NHF132‐*cumate*‐TAG, and NHF132‐kasop*‐TAG. Plus and minus signs indicate cumate inducer added or not added. Bar graphs with error bars show means ± SD (*n* = 3). Statistical significance was assessed by Student's *t*‐test (E) or one‐way ANOVA (G−I). *p < 0.05.

The ddTAG strategy is a metabolic engineering approach that dynamically regulates intracellular TAG metabolism to enhance the production of secondary metabolites such as avermectin.^[^ ^9^ ^]^ Based on our previous work, we constructed two plasmids, pCu‐TAG (**Figure** **5**A) and pKas‐TAG (Figure 5B). We employed the strong promoter kasop* and the inducible promoter *cumate* to regulate the expression of *sco6196*, encoding a long‐chain fatty acid‐CoA ligase, with the aim of increasing spinosad production. By controlling TAG degradation into fatty acids and further into acetyl‐CoA, this enzyme may redirect carbon flux from intracellular TAG and exogenous substrates to secondary metabolite synthesis, thereby boosting target product yield (Figure 5C).

We first used thin‐layer chromatography (TLC) to analyze intracellular TAG levels in *Sa. spinosa* at various fermentation timepoints. The results showed that intracellular TAG and PL levels were high at 2 days but decreased markedly during fermentation (Figure 5D), providing the basic conditions for applying the ddTAG strategy. Subsequently, we constructed recombinant strains NHF132‐kasop*‐TAG and NHF132‐*cumate*‐TAG via conjugative transfer. Fermentation results showed that NHF132‐kasop*‐TAG exhibited poor growth and much lower spinosad production, possibly due to the kasop* promoter initiating TAG conversion prematurely, thereby affecting strain growth and target production (Figure 5E,I). We then performed fermentation validation on NHF132‐*cumate*‐TAG and optimized the induction conditions (Figure 5F). The results revealed that induction at day 1 with 50 µm cumate was optimal. Spinosad production increased 23.7% from 279.5 to 345.7 mg L^−1^ (Figure 5G). qPCR analysis showed that *sco6196* expression was considerably higher in the cumate‐induced group than in the uninduced control (Figure 5H). These results indicated that the ddTAG strategy can modestly increase spinosad production in NHF132. However, this enhancement ratio still fails to meet the requirements of industrial production. Additionally, large growth differences among actinomycete batches negatively influence the effectiveness and reproducibility of the ddTAG strategy, for which the induction timing is crucial.

### 2.5 Introduction of an Artificial NCM Pathway in *Sa. Spinosa*

**Figure 6:**
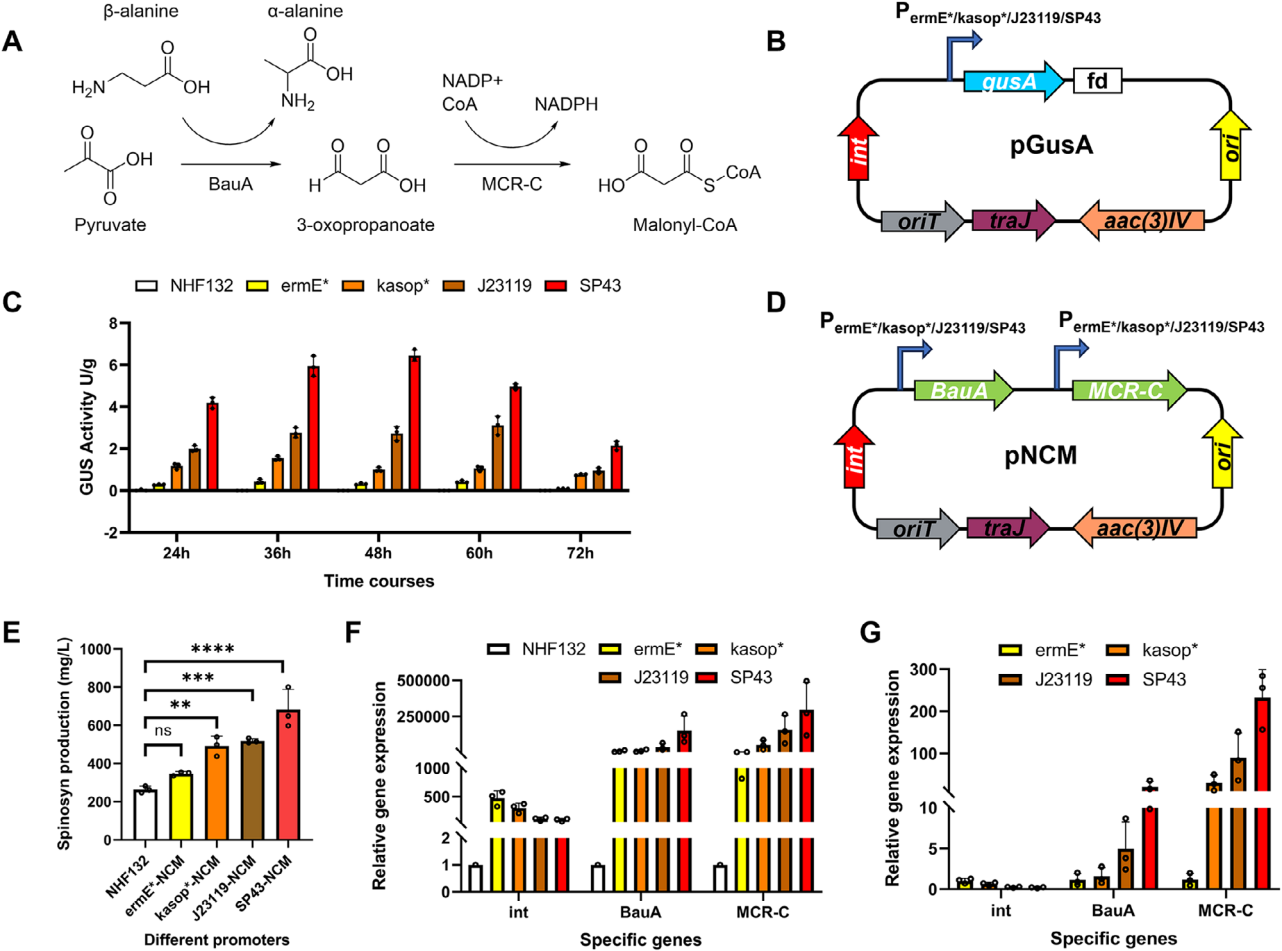
The NCM strategy for spinosad overproduction in *Sa. spinosa* NHF132. A) Schematic diagram of the NCM strategy that converts C_3_–C_3_ via 3‐oxopropanoate without CO_2_ release, carbon loss, or ATP consumption. B) The *gusA* reporter plasmid pGusA. fd, central terminator from bacteriophage fd. C) Promoter strengths of the selected native and reference promoters in *Sa. spinosa*, as assessed from GUS reporter protein activity. D) Plasmid pNCM with different promotors (ermE*/kasop*/J23119/SP43). E) Spinosad (spinosyn A/D) production in wild‐type strain NHF132 and engineered strain NHF132‐ermE*/kasop*/J23119/SP43‐NCM under the control of different promotors. F) qPCR analysis of NCM‐related genes in NHF132 and NHF132‐ermE*/kasop*/J23119/SP43‐NCM in fermentation broth on day 6. G) qPCR analysis of NCM‐related genes in NHF132‐ermE*/kasop*/J23119/SP43‐NCM (relative to NHF132‐ermE*‐NCM). Bar graphs with error bars show means ± SD (*n* = 3). Statistical significance was assessed by one‐way ANOVA. **p < 0.01; ***p < 0.001; ****p < 0.0001; ns, not significant.

Production of spinosad, a polyketide derived from acyl‐CoA, is closely related to intracellular levels of acetyl‐CoA, malonyl‐CoA, and similar molecules.^[^ ^14^ ^]^ Natural acyl‐CoA is mainly synthesized via the ACC pathway (Figure 1C), which is accompanied by carbon loss and complex regulation, thereby limiting spinosad synthesis. The artificial NCM pathway efficiently synthesizes the central metabolite malonyl‐CoA and its derived secondary metabolites (**Figure** **6**A). Replacing the natural ACC pathway with an artificial NCM pathway addresses the low efficiency, high energy consumption, and metabolic regulation issues of traditional synthesis routes, enhancing spinosad production in *Sa. spinosa*.^[^ ^15^ ^]^

Compared with the ermE*‐controlled NCM pathway, the kasop*‐controlled NCM pathway is more effective for spinosad production. Consequently, this study first mined strong promoters in *Sa. spinosa*. Using the β‐glucuronidase (gusA) reporter system, we evaluated the strength of several promoters including ermE*, kasop*, *J23119*, and *SP43* (Figure 6B). We found that the strength of *SP43* was 4–6 times that of kasop* (Figure 6C).

Based on these findings, we incorporated the NCM‐related genes *BauA* and *MCR‐C* into the pSET152 vector, placing them under the control of different promoters to enhance spinosad production. Using primers with varying promoter strengths, we constructed several NCM expression vectors, including pSET152‐ermE*‐*BauA*‐ermE*‐*MCR‐C*, pSET152‐kasop*‐*BauA*‐SPL42‐*MCR‐C*, pSET152‐J23119‐*BauA*‐J23119‐*MCR‐C*, and pSET152‐SP43‐*BauA*‐*SP43*‐*MCR‐C*, listed in increasing strength (Figure 6D).

Fermentation validation of the resulting conjugants showed that NCM‐containing recombinants consumed more medium than the wild‐type strain. In shake flask cultures, the time for glucose depletion advanced from day 2 to day 1. Cultures rapidly became less viscous, indicating rapid nutrient metabolism. The fermentation cycle was shortened from 8 to 6 days. Spinosad production also increased substantially, with stronger promoters leading to greater improvements. For instance, NHF132‐ermE*‐NCM increased from 263.6 to 347.2 mg L^−1^ (+31.7%), NHF132‐kasop*‐NCM to 492.0 mg L^−1^ (+86.6%), NHF132‐J23119‐NCM to 518.07 mg L^−1^ (+96.51%), and NHF132‐*SP43*‐NCM to 682.2 mg L^−1^ (+158.8%; Figure 6E). qPCR results showed expression level differences for *BauA* and *MCR‐C* under various promoters, confirming that introducing the NCM pathway markedly boosted spinosad production and was positively correlated with NCM gene expression (Figure 6F,G). The above results indicate that the supply of key acyl‐CoA precursors is one of the critical factors determining the production of spinosad.

### 2.6 Competitive BGC Knockout in *Sa. spinosa* Using an Endogenous Type I CRISPR/Cas

**Figure 7:**
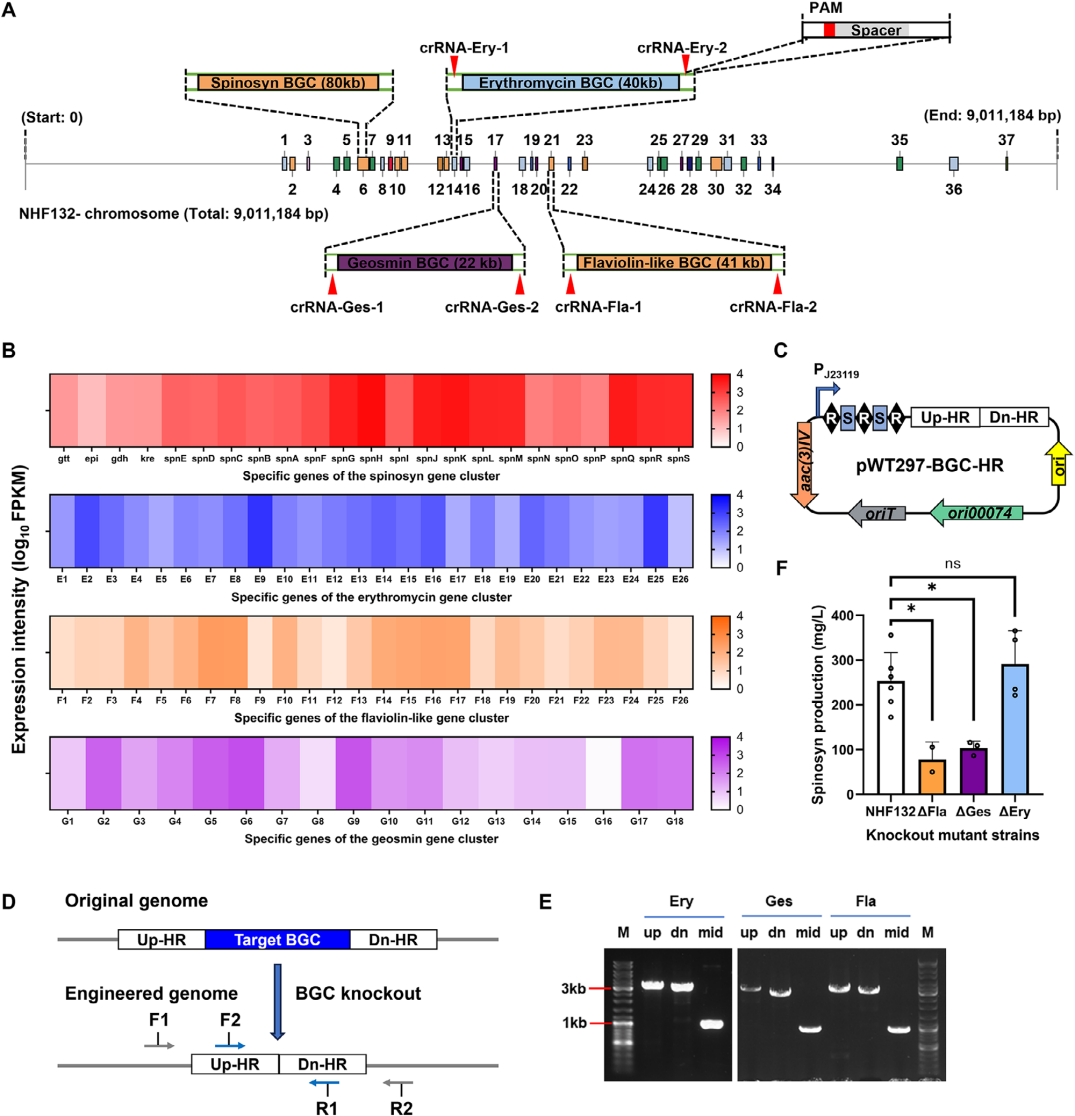
Knockout of competitive BGCs for spinosad overproduction in *Sa. spinosa* NHF132. A) AntiSMASH analysis of *Sa. spinosa* NHF132 and genomic locations of the four selected BGCs (Spn, Ery, Fla‐like, and Ges) in the chromosome. B) RNA sequencing (RNA‐seq) expression profiles of the four selected BGCs in NHF132 fermentation broth on day 4. Specific genes were predicted by antiSMASH and renumbered. C) Editing plasmid of pWT297‐BGC‐HR containing donor DNA and an artificial CRISPR array. *ori00074*, *Sa. spinosa*‐specific replication origin. D) Schematic diagram of BGCs knockout via endogenous CRISPR‐mediated homologous recombination. E) PCR verification of NHF132‐ΔEry/ΔFla/ΔGes. Up, dn, and mid are the PCR products amplified with F1/R1, F2/R2, and F2/R1 (sequences in Table S3, Supporting Information), respectively. M, molecular markers. F) Spinosad (spinosyn A/D) production in wild‐type strain NHF132 and engineered strain NHF132‐ΔEry/ΔFla/ΔGes. Bar graphs with error bars show means ± SD (*n* = 3). Statistical significance was assessed by one‐way ANOVA. *p < 0.05; ns, not significant.

Elimination of non‐target BGCs is a classic strategy to enhance NP yields. It can decrease precursor competition and metabolic load, and improve product purity.^[^ ^47^, ^48^ ^]^ Disrupting non‐target metabolic pathways can also create versatile chassis strains suitable for the production of multiple products.^[^ ^39^ ^]^ In this study, we used antiSMASH to analyze the genome of *Sa. spinosa* NHF132. We identified 37 potential BGCs, including 10 PKS (4 type I PKS, 4 type III PKS, and 2 PKS‐like), 7 terpene, and 9 NRPS clusters (Table S10, Supporting Information), and located the target spinosad BGC (Region 6; **Figure** **7**A). Among them, regions 3, 6, 14, 15, 17, 20, 21, 26, 31, and 37 exhibit high similarity to BGCs with known functions (>70%). Additionally, to minimize effects on strain growth, we excluded region 26 (associated with coelibactin, a potential zinc carrier, knockout of which may affect sporulation in Streptomyces) and region 37 (related to ectoine, crucial for regulating cellular osmotic pressure). A *Sa. erythraea* chassis with erythromycin (Ery) BGC knockout and enhanced heterologous expression of polyketide antibiotics was previously reported,^[^ ^47^ ^]^ and the butenyl‐spinosad biosynthesis route in *Sa. pogona* was optimized by flaviolin‐like (Fla‐like) BGC deletion.^[^ ^48^ ^]^ Meanwhile, deletion of the geosmin (Ges) BGC was reported to increase spinosad production and improve the tail gas odor.^[^ ^49^ ^]^ Guided by these previous studies, we finally selected three BGCs, Ery (region 14), Fla‐like (region 17), and Ges (region 21), with substantial transcription levels for knockout, aiming to boost the yield of the target compound spinosad (Figure 7B).

Using the endogenous type I CRISPR/Cas system and homologous recombination, we constructed three knockout plasmids, pWT297‐Ery‐HR, pWT297‐Fla‐HR, and pWT297‐Ges‐HR (Figure 7C), and obtained knockout strains NHF132‐ΔEry, NHF132‐ΔFla, and NHF132‐ΔGes via conjugative transfer (Figure 7D). PCR confirmed the genotype of NHF132‐ΔEry, showing that the complete Ery gene cluster was knocked out (Figure 7E). Similarly, we verified NHF132‐ΔFla and NHF132‐ΔGes. Fermentation tests revealed that spinosad yields decreased in NHF132‐ΔFla and NHF132‐ΔGes, while the increase in NHF132‐ΔEry was not significant (from 253.0 to 291.7 mg L^−1^; Figure 7F). In conclusion, knockout of Ery did not significantly enhance spinosad production. However, knockout of Fla‐like resulted in a marked yield decrease, differing greatly from previous reports.^[^ ^48^ ^]^ This discrepancy may be attributable to previously unrecognized functions within region 17 of NHF132, or to intrinsic variations between *Sa. spinosa* and *Sa. pogona*. Geosmin, a terpenoid odorant that is responsible for the smell of fresh earth following rain,^[^ ^50^ ^]^ was knocked out in actinomycetes for the first time to test its impact on NP yields. In *Streptomyces*, it may serve as an environmental signaling molecule to respond to environmental changes, which explains why Ges knockout lowered the spinosad yield.

### 2.7 Combined Metabolic Engineering Strategies for Spinosad Production

From the prediction targets of Spn‐GEM (Table S9, Supporting Information), we found that spinosad biosynthesis is closely related to amino acid, nucleotide, and carbohydrate metabolism. Upregulation of pyruvate kinase and downregulation of isocitrate lyase indicated that we need to maximize the use of precursors for spinosad production without affecting basic metabolism, consistent with the biosynthetic pathway of spinosad (Figure 1C). We tested various strategies that can be categorized into three classes: precursor supplementation, BGC overexpression, and chassis reconstruction. Among them, precursors could be further divided into rhamnose and acyl‐CoA. To enhance spinosad production further, we integrated the aforementioned strategies, such as combining BGC doubling with rhamnose overexpression, BGC doubling with NCM, and ddTAG with NCM.

**Figure 8:**
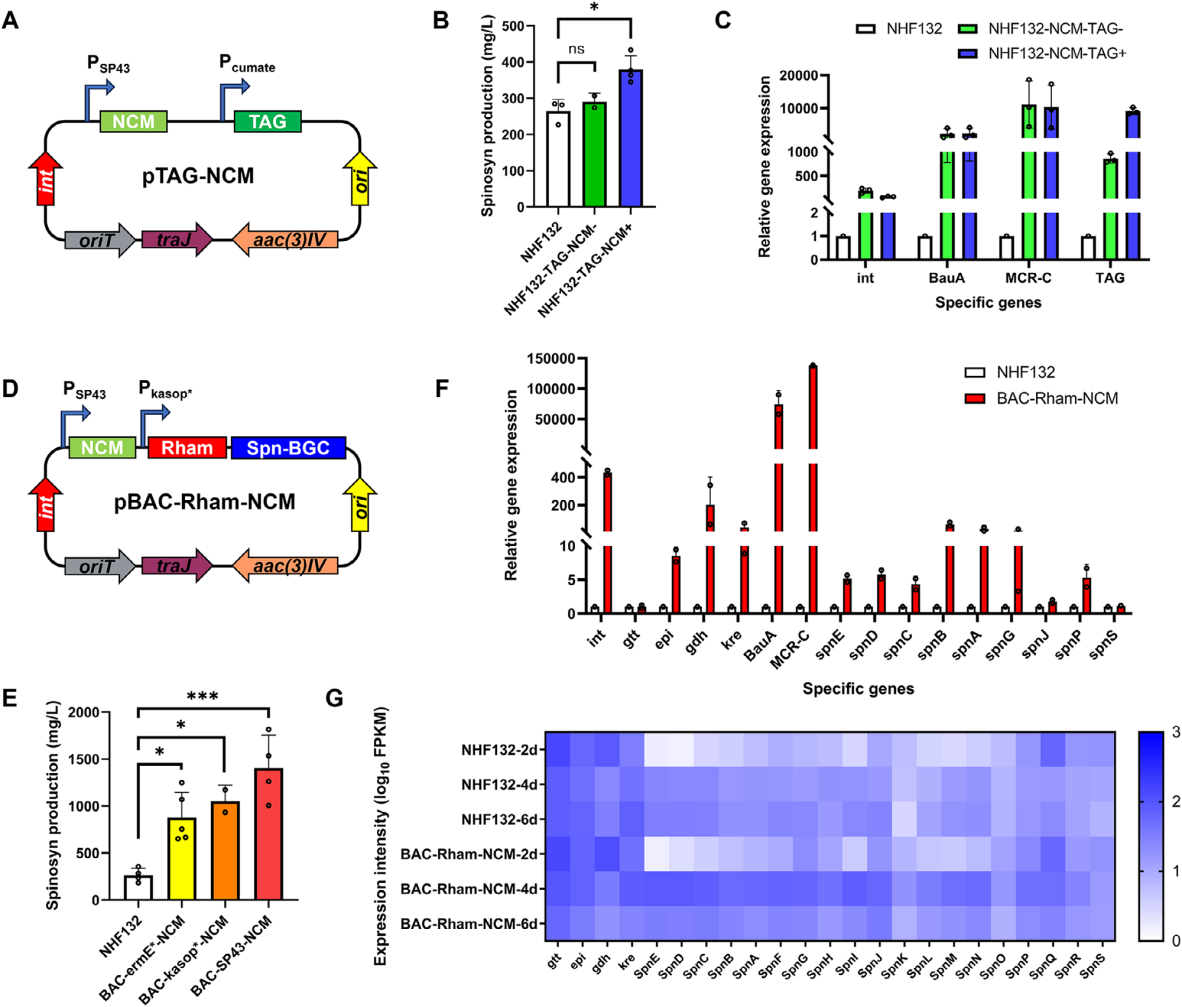
Combined strategies for spinosad overproduction in *Sa. spinosa* NHF132. A) The pTAG‐NCM vector incorporating both ddTAG and NCM strategies. B) Spinosad (spinosyn A/D) production in wild‐type strain NHF132 and engineered strain NHF132‐TAG‐NCM. Plus and minus signs indicate cumate inducer addition or no addition. C) qPCR analysis of related genes in NHF132 and NHF132‐TAG‐NCM in fermentation broth on day 6. D) The pBAC‐Rham‐*SP43*‐NCM vector incorporating the NCM pathway, the Rham biosynthetic module, and the complete spinosad BGC. E) Spinosad (spinosyn A/D) production in wild‐type strain NHF132 and engineered strain NHF132‐BAC‐(ermE*/kasop*/SP43)‐NCM. F) qPCR analysis of rhamnose, NCM, and BGC‐related genes in NHF132 and NHF132‐BAC‐*SP43*‐NCM in fermentation broth on day 6. G) RNA‐seq analysis of rhamnose and BGC‐related genes in NHF132 and NHF132‐BAC‐*SP43*‐NCM during the fermentation time‐course. Bar graphs with error bars show means ± SD (*n* = 3). Statistical significance was assessed by one‐way ANOVA. *p < 0.05; ***p < 0.001; ns, not significant.

As the ddTAG strategy and NCM pathway both aim to enhance precursor acyl‐CoA flux, we initially constructed the pTAG‐NCM vector incorporating both ddTAG and NCM strategies to test their synergistic potential (**Figure** **8**A). However, fermentation results showed that recombinant strain NHF132‐TAG‐NCM exhibited only a 29.1% increase in spinosad production (from 264.6 to 351.2 mg L^−1^), similar to the outcome of the standalone ddTAG strategy (Figure 8B). Additionally, qPCR results revealed that expression levels of genes related to both TAG and NCM strategies were considerably elevated in recombinant strain NHF132‐TAG‐NCM (Figure 8C), thereby confirming the effective expression of these genes. We hypothesized that the efficacy of the NCM strategy depends on efficient carbon source utilization, while metabolic flux redirection by ddTAG may reduce carbon availability, thereby hindering the effectiveness of the NCM pathway.

Given that the NCM strategy outperformed ddTAG in initial tests, we chose to combine rhamnose overexpression, BAC doubling, and the artificial NCM pathway to systematically enhance spinosad production. Using RedET methods, we constructed the pBAC‐Rham‐(ermE*/kasop*/SP43)‐NCM vector incorporating the NCM pathway, the Rham biosynthetic module, and the complete spinosad BGC (Figure 8D). Remarkably, fermentation analysis revealed a synergistic enhancement in spinosad production by NHF132‐BAC‐(ermE*/kasop*/SP43)‐NCM, with an enhancement surpassing the combined individual effects. Notably, strain NHF132‐BAC‐*SP43*‐NCM achieved a spinosad titer of 1405.4 mg L^−1^ in shake flask cultures, a 429.2% increase over the wild‐type strain (Figure 8E). qPCR results also indicated a considerable increase in the expression levels of BGC, rhamnose, and NCM‐related genes in the recombinant strains (Figure 8F). Moreover, whole‐transcriptome RNA‐seq was performed on the wild‐type strain NHF132 and the engineered strain NHF132‐BAC‐*SP43*‐NCM during the fermentation time‐course. The expression of rhamnose and BGC‐related genes was higher in the high‐yield strain relative to the wild‐type (Figure 8G), corroborating the qPCR data. This synergistic enhancement suggests that complex metabolic processes may involve multiple constraints, while simply enhancing a single precursor or overexpressing a synthetic gene may not effectively increase target compound production. This further highlights the effectiveness of model‐guided systems metabolic engineering.

### 2.8 Optimization of the Medium and Fermentation Process for *Sa. Spinosa Culture*

Optimizing the medium and/or fermentation conditions is an effective strategy for enhancing NP yields. Adjusting nutritional components, refining medium composition, controlling physicochemical properties, and improving fermentation settings can all effectively boost target product formation. Previous studies demonstrated that optimizing carbon and nitrogen sources, adding precursors, and improving fermentation conditions can effectively increase spinosad production.^[^ ^24^, ^51^ ^]^

**Figure 9:**
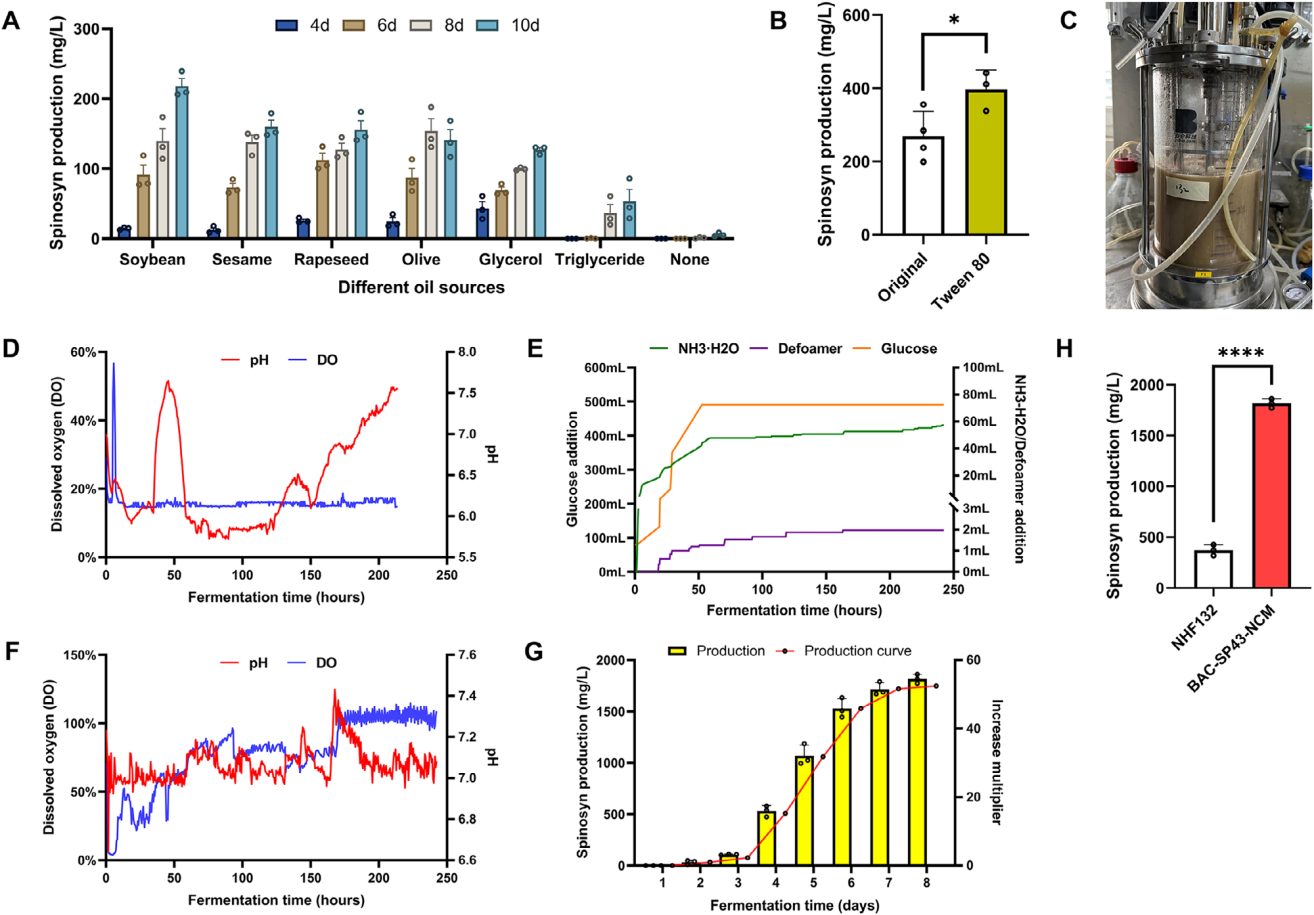
Medium and fermentation process optimization for spinosad overproduction in *Sa. spinosa* NHF132. A) Spinosad (spinosyn A/D) production in wild‐type strain NHF132 with different plant oil components. B) Spinosad (spinosyn A/D) production in wild‐type strain NHF132 with and without 400 mg L^−1^ Tween 80 added. C) Bioreactor equipped with pH, DO, and temperature sensors, and feeding devices. D) Batch fermentation parameter monitoring of NHF132‐BAC‐*SP43*‐NCM. E) Feeding curve during NHF132‐BAC‐*SP43*‐NCM fermentation. F) Fed‐batch fermentation parameter monitoring of NHF132‐BAC‐*SP43*‐NCM after feeding optimization. G) Spinosad (spinosyn A/D) production during NHF132‐BAC‐*SP43*‐NCM fermentation after feeding optimization. H) Spinosad (spinosyn A/D) production in wild‐type strain NHF132 and engineered strain NHF132‐BAC‐*SP43*‐NCM after medium and fermentation process optimization. Bar graphs with error bars show means ± SD (*n* = 3). Statistical significance was assessed by Student's *t*‐test (B,H) or one‐way ANOVA (A, G). *p < 0.05; ****p < 0.0001.

In this study, we first optimized the plant oil components in the carbon source. Plant oils, primarily composed of TAGs, are broken down into fatty acids and glycerol during microbial fermentation, and are further metabolized into acetyl‐CoA.^[^ ^9^ ^]^ We tested various carbon sources including soybean oil, sesame oil, rapeseed oil, olive oil, glycerol, and TAGs (**Figure** **9**A). Fermentation results showed that soybean oil is an optimal carbon source, increasing spinosad production of wild‐type NHF132 from 188.2 to 239.3 mg L^−1^, achieving a 27.2% improvement. Additionally, supplementing the medium with 400 mg L^−1^ Tween 80 raised spinosad production from 269.3 to 397.1 mg L^−1^, representing a 47.5% increase (Figure 9B).

To simulate industrial production conditions, we cultivated the high‐yielding strain NHF132‐BAC‐*SP43*‐NCM in a bioreactor (Figure 9C). Fermentation process monitoring revealed a sharp pH drop around day 2 of cultivation (Figure 9D). Under batch culture conditions, the culture was exposed to an acidic environment (pH < 6.0) during late fermentation stages, which may adversely affect *Sa. spinosa* growth and spinosad production. In this study, we optimized fermentation conditions by feeding glucose in the early fermentation stage to enhance mycelium accumulation and reduce carbon catabolite repression, while feeding aqueous ammonia to adjust pH and adding defoamer to enhance mass transfer and boost dissolved oxygen (DO) levels (Figure 9E). Through fed‐batch culture, the pH was maintained at ≈7, and DO was kept at a relatively high level (>50%; Figure 9F). Based on the optimized medium conditions, we tested the fermentation of NHF132‐BAC‐*SP43*‐NCM and achieved a spinosad titer of 1816.8 mg L^−1^ (Figure 9G), a 29.3% increase over the original fermentation conditions and a 553.3% increase over the original starting strain *Sa. spinosa* NHF132 (Figure 9H).

## 3 Conclusion

Current metabolic engineering strategies for enhancing spinosad production vary due to differences in parent strain and culture conditions, making it difficult to compare their effectiveness directly. In this study, we selected *Sa. spinosa* NHF132 as the parent strain and evaluated multiple metabolic engineering strategies for enhancing spinosad production, including rhamnose overexpression, BGC overexpression, a ddTAG strategy, the NCM pathway, knockout of competitive BGCs, medium optimization, and fermentation process optimization. Under single‐factor conditions, except for knockout of competitive BGCs, all strategies increased spinosad production. Furthermore, modeling analysis revealed that spinosad biosynthesis is a complex process influenced by primary and secondary metabolism (Table S9, Supporting Information), such as glycometabolism, amino acid metabolism, fatty acid metabolism, and specific glycoside synthesis. Enhancing the supply of direct precursors and boosting expression of biosynthetic genes can improve target compound synthesis efficiency and production.

This study innovatively integrated rhamnose overexpression, complete spinosad BGC doubling, and the NCM artificial pathway. This systematic metabolic engineering enhanced precursor flux and overexpressed key synthetic enzymes, achieving high‐efficiency spinosad synthesis and a remarkable 429.2% increase in spinosad yield, from 265.6 to 1405.4 mg L^−1^, surpassing the combined effects of the two individual strategies. Spn‐GEM analysis suggested that single‐factor modifications alone may be insufficient to achieve desired outcomes (Figure 1C). For example, intracellular rhamnose accumulation depends on glucose conversion, synthetic gene expression, and bacterial growth‐related consumption. Moreover, even with increased rhamnose flux, spinosad yield cannot be substantially improved if aglycone levels are inadequate. This explains why previous metabolic engineering strategies often failed to increase spinosad production in high‐yielding strains, while the systematic combinatorial strategy in this study markedly enhanced spinosad yield.

Furthermore, we monitored the fermentation of the high‐yielding strain NHF132‐BAC‐*SP43*‐NCM in a bioreactor. Through fed‐batch cultivation with precise pH, DO, and glucose control, spinosad production increased by 29.3%, reaching 1816.8 mg L^−1^, a 553.3% improvement over wild‐type NHF132. This shows that combining precursor supplementation, gene overexpression, and medium optimization via systematic metabolic engineering can effectively enhance spinosad production, and may offer a promising strategy for improving the yields of other NPs.

## 4 Experimental Section

### Bacterial Strains and Culture Conditions

All bacterial strains utilized in this study are listed in Table S1 (Supporting Information). *Escherichia coli* DH10b and *E. coli* S17‐1 were cultured at 37 °C in Luria–Bertani (LB) broth or on agar supplied with appropriate antibiotics. *Sa. spinosa* strains were grown at 30 °C in 2CMC medium (modified HAUCM medium, pH 7.2) consisting of 10 g of soluble starch, 1 g of NaCl, 2 g of tryptone, 2 g of casein amino acids, 2 g of (NH_4_)2SO_4_, 1 g of K_2_HPO_4_, 2 g of MgSO_4_, 1 mg of FeSO_4_•7H_2_O, 1 mg of ZnSO_4_•7H_2_O, 2 g of CaCO_3_, and 20 g of agar in 1 L of distilled water; 1.5 mL of sterilized MgCl_2_ (2.5 M) was added to each 100 mL of medium before use.^[^ ^52^ ^]^ This medium was also employed for intergeneric conjugation between *E. coli* S17‐1 and *Sa. spinosa*.^[^ ^53^ ^]^ For spinosad fermentation, *Sa. spinosa* strains were incubated on an orbital shaker at 30 °C with shaking at 220 rpm in seed medium (30 g of tryptone soya broth (TSB), 3 g of yeast extract, 3 g of Lab‐Lemco powder, 2.5 g of corn steep powder, and 10 g of glucose) for 2 days. Subsequently, the culture was inoculated into fermentation medium (30 g of soluble starch, 5 g of yeast extract, 15 g of soybean cake powder, 10 g of cottonseed meal, 15 g of corn steep powder, 5 mL of soybean oil, 5 g of CaCO_3_, and 40 g of glucose) at a 10% inoculum and cultured for 6−8 days.

### DNA Cloning and Plasmid Construction

All plasmids utilized in this study are listed in Table S2 (Supporting Information). Mini‐preparations of plasmid DNA, transformation of *E. coli*, and PCR amplification were conducted as described previously.^[^ ^54^ ^]^ Genomic and plasmid DNA isolation and manipulation in actinomycetes were performed as outlined previously.^[^ ^55^ ^]^ For expression vectors, pSET152 was employed for smaller insert fragments and pBAC2015 was selected for larger inserts, such as gene clusters.^[^ ^41^, ^56^ ^]^ Both plasmids can be integrated into genomic DNA via phiC31‐mediated site‐specific integration. Expression vectors were individually transformed into *Sa. spinosa* NHF132 by *E. coli‐Sa. spinosa* biparental conjugation and verified through antibiotic resistance screening and PCR analysis to select the correct conjugants. Primers used for plasmid construction and PCR verification are listed in Table S3 (Supporting Information). Agarose gel electrophoresis verification results for expression plasmids and engineered strains are provided in Figures S1 and S2 (Supporting Information).

### DNA Cloning and Plasmid Construction—Plasmids Carrying Rhamnose‐Related Genes

The four rhamnose synthetic genes (*gtt*, *epi*, *gdh*, and *kre*) were amplified from NHF132 genomic DNA using primer pairs gtt‐HR‐F/R, epi‐HR‐F/R, and gdh‐HR‐F/kre‐HR‐R. The *spnG, H, I, and K* genes were amplified from NHF132 genomic DNA using primer pairs spnGHI‐HR‐F/R and spnK‐HR‐F/R. PCR products were cloned into pSET152 to generate pRham‐GHIK by homologous recombination. The strong, constitutive promoter kasop* was placed upstream of the above genes.

### DNA Cloning and Plasmid Construction—Plasmids Carrying Spinosad BGCs

The pBAC‐Spn plasmid carrying the complete spinosyn gene cluster was derived from pBAC2015‐81kb‐J1074 by replacing chloramphenicol resistance (Chl^R^) with apramycin resistance (Apr^R^).^[^ ^57^ ^]^

### DNA Cloning and Plasmid Construction—Plasmids Carrying the ddTAG System

To construct the ddTAG strategy plasmid, *cumate‐sco6196* and kasop*‐sco6196 sequences were amplified from pCu‐SCO6196 and pKas‐SCO6196 plasmid DNA using primer pairs Cu‐*LacZa*‐HR‐F/*sco6196*‐HR‐R and kasop*‐*LacZa*‐HR‐F/*sco6196*‐HR‐R, respectively.^[^ ^9^ ^]^ The two amplified fragments were separately cloned into pSET152 using a One Step Cloning Kit (ABclonal, China), yielding recombinant plasmids pCu‐TAG and pKas‐TAG.

### DNA Cloning and Plasmid Construction—Plasmids Carrying Artificial NCM Pathway Genes

To construct the NCM pathway plasmid, *BauA* and *MCR‐C* sequences were amplified from pSET152‐kasop*‐*BauA*‐SPL42‐*MCR‐C* plasmid DNA using primer pairs Lac‐*SP43*‐F/*BauA*‐*SP43*‐R and *BauA*‐*SP43*‐F/*MCR‐C*‐*SP43*‐R, respectively.^[^ ^15^ ^]^ The strong promoter *SP43* was directly incorporated into primers. The two amplified fragments were cloned into pSET152 using a One Step Cloning Kit (ABclonal), yielding recombinant plasmid pSET152‐*SP43*‐*BauA*‐*SP43*‐*MCR‐C*. By synthesizing primers carrying promoters of varying strengths, pSET152‐*J23119*‐*BauA*‐*J23119*‐*MCR‐C* plasmids were obtained in the same way.

### DNA Cloning and Plasmid Construction—Plasmid Carrying NCM Pathway Genes and the ddTAG System

To construct the plasmid incorporating both ddTAG and NCM strategies, the *cumate‐sco6196* sequence was amplified from pCu‐TAG plasmid DNA using primer pair TAG‐NCM‐HR‐F/R. The PCR product was cloned into pSET152‐*SP43*‐*BauA*‐*SP43*‐*MCR‐C* to generate pTAG‐NCM by homologous recombination.

### DNA Cloning and Plasmid Construction—Plasmids Carrying the BGC and Other Genes

To combine the BGC overexpression strategy with other approaches, the RedET direct cloning method was employed to manipulate the large BAC plasmid as described previously.^[^ ^58^ ^]^ *Amp‐Rham* gene sequences were amplified from the pRham‐Amp plasmid DNA using primer pairs Rham‐NCM‐HR‐F/Rham‐BAC‐HR‐R. Primers SP43‐NCM‐BAC‐HR‐F/NCM‐Rham‐HR‐R (each with 20 bp BAC homologous arms at both ends) were used to amplify the *SP43*‐*BauA*‐*SP43*‐*MCR‐C* fragment from pSET152‐*SP43*‐*BauA*‐*SP43*‐*MCR‐C* plasmid DNA. To modify the pBAC‐Spn plasmid, DH10b‐pBAC‐Spn competent cells were prepared and transformed with pSC101 carrying the RedET element. Recombinant strains were selected with Apr and tetracycline (Tet). Next, DH10b‐pBAC‐pSC101 was incorporated into competent cells, which were subsequently transformed with the target *SP43*‐*BauA*‐*SP43*‐*MCR‐C* and the *Amp‐Rham* fragment. Strains carrying the target plasmid pBAC‐Rham‐(*SP43*)‐NCM were obtained through selection with Apr and ampicillin. By synthesizing primers carrying promoters of varying strengths, plasmids pBAC‐Rham‐(ermE*)‐NCM and pBAC‐Rham‐(kasop*)‐NCM were obtained in the same way. The correct plasmids were verified using primer pairs BAC‐v‐F/NCM‐v‐R, NCM‐v‐F/Rham‐v‐R, and Rham‐v‐F/BAC‐v‐R.

### Establishment and Optimization of the *Sa. spinosa* Conjugative Transfer System

Mycelium was employed as recipient cells. A specific quantity of spores was inoculated into a 30 mL TSB liquid medium at 30 °C with shaking at 220 rpm for 48 h. The culture was then transferred to fresh TSB liquid medium at a 10% inoculum volume and cultured for 6 h to prepare recipient cells for conjugative transfer. For donor cells, *E. coli* cells harboring recombinant plasmid were cultured in LB medium at 37 °C with shaking at 220 rpm for 12−16 h. This culture was then transferred to fresh LB liquid medium at a 1% inoculum volume and cultured to an optical density at 600 nm (OD_600)_ of 0.6−0.8. Recipient and donor cells were washed twice with fresh TSB and LB liquid medium, respectively, then mixed thoroughly at a ratio of 10^8^:10^8^. The mixed bacterial solution was spread on non‐anti‐2CMC medium and incubated at 30 °C for 16−20 h. The plate was overlaid with a solution of nalidixic acid (NA) and Apr at a final concentration of 50 µg mL^−1^ as screening conditions. After incubation at 30 °C for 7−14 days, growth of white conjugants was observed and verified.

### Screening for High‐Activity Promoters Using the *GusA* Reporter System

The *gusA* gene was amplified from pBBR‐*gusA* plasmid DNA using primers *gusA*‐F/R.^[^ ^59^ ^]^ The PCR product was cloned into pSET152 via homologous recombination to generate the pSET152‐*gusA* vector. A series of promoters, including ermE*, kasop*, *J23119*, and *SP43* ^[^ ^60^ ^]^ (Table S5, Supporting Information) were synthesized and cloned into the pSET152‐*gusA* vector to generate pSET152‐kasop*‐*gusA*, pSET152‐ermE*‐*gusA*, pSET152‐*J23119*‐*gusA*, and pSET152‐*SP43*‐*gusA*. After obtaining the corresponding conjugants, monoclonal isolates were inoculated into TSB medium for initial cultivation and subsequently subcultured into fresh TSB at a 10% inoculum volume. The entire process was conducted with three biological replicates. Samples were collected at 24, 36, 48, 60, and 72 h, with 2 mL of culture harvested each time. A 1 mL volume of each sample was used for GUS activity measurement, while the remaining 1 mL was used to determine dry cell weight as an indicator of growth. GUS enzymatic assay procedures were conducted as previously described.^[^ ^61^ ^]^

### Deletion of Ery, Fla‐Like, and Ges Gene Clusters

For deletion of the Ery gene cluster, the possible gene cluster region between S1GL005577 and S1GL005619 was selected as the knockout target, using the CRISPR‐Cascade‐Cas3‐based approach.^[^ ^62^ ^]^ A double‐stranded DNA encoding a pair of guide RNA scaffolds was obtained by gene synthesis. This DNA fragment was cloned into the pWT297 vector via the *Bam*HI and *Sal*I restriction sites to generate pWT297‐Ery. The 2.3 kb upstream homologous arm (UHA) and 2.2 kb downstream homologous arm (DHA) sequences were amplified from *Sa. spinosa* NHF132 genomic DNA using primer pairs Ery‐UHR‐F/Ery‐UHR‐R and Ery‐DHR‐F/Ery‐DHR‐R, respectively. These PCR products were cloned into pWT297‐Ery at the *Eco*RV site to generate the Ery gene cluster replacement vector pWT297‐Ery‐HR.

For deletion of the Fla‐like gene cluster, the possible gene cluster region (including a putative type III PKS domain) between S1GL004638 and S1GL004688 was selected as the knockout target. For deletion of the Ges gene cluster, the possible gene cluster region (including a putative terpene synthase) between S1GL005196 and S1GL005217 was selected as the knockout target. Using the same method, Fla‐like and Ges gene cluster replacement vectors pWT297‐Fla‐HR and pWT297‐Ges‐HR, respectively, were constructed.

The pWT297‐Ery‐HR, pWT297‐Fla‐HR, and pWT297‐Ges‐HR constructs were separately transformed into *Sa. spinosa* NHF132 by *E. coli*‐*Sa. spinosa* biparental conjugation. Independent Apr‐resistant conjugants were streaked onto solid 2CMC medium, containing 50 µg mL^−1^ Apr (for selection against the vector backbone) and 30 mg mL^−1^ nalidixic acid (for selection against *E. coli*), then grown in the dark for 5 days. Apr‐resistant colonies were initially cultured in TSB liquid medium without Apr for 2−3 days. Subsequently, these colonies were spread and diluted on 2CMC medium without Apr and grown for 5−8 days. Finally, colonies were replicated onto plates with or without Apr to confirm plasmid loss. Genomic DNA of single Apr‐sensitive colonies was extracted and used to screen for gene replacement mutants via PCR verification. The Ery BGC knockout mutant, designated as *Sa. spinosa* NHF132‐ΔEry, was confirmed by PCR with primer pairs 132‐Ery‐up‐v‐F/Ery‐DHR‐v‐R and Ery‐UHR‐v‐F/132‐Ery‐dn‐v‐R and DNA sequence analysis. Similarly, the Fla‐like BGC knockout mutant (*Sa. spinosa* NHF132‐ΔFla) was confirmed by PCR using primer pairs 132‐Fla‐up‐v‐F/132‐Fla‐dn‐v‐R and DNA sequence analysis, and the Ges BGC knockout mutant (*Sa. spinosa* NHF132‐ΔGes) was confirmed by PCR using primer pairs 132‐Ges‐up‐v‐F/132‐Ges‐dn‐v‐R and DNA sequence analysis.

### Chromatographic Analysis of Spinosad Production Using High‐Performance Liquid Chromatography (HPLC)

After fermentation, cultures harvested on days 2, 4, 6, and 8 were each extracted using three volumes of acetonitrile, followed by vortex oscillation for 2 min and sonication for 30 min. The mixtures were then incubated at 4 °C for 2−3 h and centrifuged at 13 000 × *g* for 10 min. The liquid phase was filtered through a 0.2 µm filter and analyzed by HPLC using an Agilent 1100 series system (Agilent Inc., Santa Clara, CA, USA) equipped with a Thermofisher‐C18 reversed‐phase column (3.0 µm, 4.6 × 150 mm). The column was eluted at a flow rate of 0.6 mL min^−1^ for 25 min with acetonitrile/0.1% formic acid aqueous solution (gradient details in Table S6, Supporting Information). Metabolites were monitored at a wavelength of 245 nm. Data were representative of three independent experiments. Spinosad standard (comprising 82% spinosyn A and 18% spinosyn D) was tested as a reference each time (Figure S3, Supporting Information). A calibration curve was constructed by plotting the peak area against known standard concentrations (Figure S6, Supporting Information). The peak area of spinosyn A and spinosyn D was used for quantification. Comparative HPLC chromatograms between engineered and parental strains are provided in Figure S7 (Supporting Information).

### Analysis of Intracellular TAGs

TAGs were purified as described previously^[^ ^63^ ^]^ with slight modifications. Cells were harvested from 500 µL of fermentation broth via centrifugation at 13 000 rpm for 1 min at −20 °C. Cells were promptly submerged in liquid nitrogen for 2 min then lyophilized using a vacuum concentrator for 30−60 min. Total lipids were extracted from 10 mg of lyophilized cells using chloroform/methanol (2:1, v/v) in a water bath at 40 °C for 3 h (for reference, 100 mg dry cells were extracted with 30 mL of organic phase). The mixture was vortexed for 1 min every 30 min throughout the extraction period. After extraction, 100−200 µL of chloroform/methanol (2:1, v/v) was added to the liquid phase to dissolve the residue after drying. TAGs and PLs were analyzed via TLC on silica gel 60 F254 plates (Merck, Germany) as previously described.^[^ ^64^ ^]^ Lipid fractions were visualized using Cu‐phosphoric acid staining and bands corresponding to TAGs and PLs were quantified using a Gel Imaging System (Tanon, China). The developing agent was hexane/ether/acetic acid (80:20:1, v/v/v). The developer was 10% copper sulfate dissolved in 8% phosphoric acid (1:1, v/v).

### RNA Preparation and qPCR

Samples were collected from fermentation broth on days 4 and 6. RNA preparation and qPCR analysis were performed as described previously.^[^ ^65^ ^]^ Primers utilized for qPCR are listed in Table S4 (Supporting Information). For transcriptional analysis in *Sa. spinosa* NHF132, the *rpL13* gene (encoding ribosomal protein L13) and the *sigA* gene (encoding RNA polymerase sigma factor SigA) served as internal controls. qPCR analysis was performed in triplicate for each transcript and repeated with three independent RNA samples. Relative expression levels of the tested genes were normalized against *rpL1*3 and *sigA*. Relative fold‐changes in expression of each gene were determined using the 2^−ΔΔC^_T_ method.^[^ ^66^ ^]^ The original qPCR data (including primers, melt temperatures, individual Cq values, and melt curves) are provided in Table S11 and Figures S8 and S9 (Supporting Information).

### Spn‐GEM Construction and Simulation

A rough GEM of *Sa. spinosa* NHF132 was constructed by incorporating the spinosad biosynthesis pathway into Sco‐GEM.^[^ ^34^ ^]^ In the rough GEM, glucose was set as the carbon source, with biomass flux fixed at increments from 20% to 80% of its theoretical maximum. For each fixed biomass flux, the spinosad production flux was maximized. Subsequently, parsimonious flux balance analysis (pFBA)^[^ ^67^ ^]^ was applied to minimize the total metabolic flux while maintaining both the fixed biomass flux and the optimal spinosad yield, resulting in the final flux distribution (Table S7, Supporting Information). Based on these distributions, reaction scores were calculated using a previously established strategy (Table S8, Supporting Information).^[^ ^33^, ^34^ ^]^

### Statistical Analysis

Results were mean values of at least three independent experiments. Statistical analyses were carried out using Microsoft Excel and GraphPad Prism. All results were expressed as means ± SD and were analyzed by Student's t‐test (two groups) or one‐way ANOVA (> 2 groups), with (*) *p < 0.05* indicating a significant difference. *p < 0.05; **p < 0.01; ***p < 0.001; ****p < 0.0001; ns, not significant.

## Conflict of Interest

The authors declare no conflict of interest.

## Author Contributions

G.‐Y.T. contributed to conceptualization, resources, supervision, funding acquisition, project administration, and review and editing. S.W. contributed to conceptualization, data curation, formal analysis, validation, investigation, visualization, and writing of the original draft. Y.L. contributed to data curation, formal analysis, validation, and investigation. Q.Z. contributed to investigation and methodology. Y.J. contributed to investigation and methodology. X.Z. contributed to investigation, methodology, and review and editing. C.Z. contributed to software, methodology, and review and editing. J.C. and W.W. contributed to review and editing. L.Z. contributed to supervision and funding acquisition.

## Supporting information



Supporting Information

Supplemental Table 7

Supplemental Table 8

Supplemental Table 9

Supplemental Table 10

Supplemental Table 11

## Data Availability

The data that support the findings of this study are available in the supplementary material of this article.
