# Supplementary material for: Model‐Guided Systematic Metabolic Engineering for Enhanced Spinosad Biosynthesis in Saccharopolyspora spinosa NHF132
Source: Adv Sci (Weinh). 2025 Sep 26;12(47):e11411. doi: 10.1002/advs.202511411 (PMC12713090; doi:10.1002/advs.202511411)
Supplement: Supplementary file 1 — Supporting Information [file ADVS-12-e11411-s002.docx]

**Supporting Information**

**Model-guided Systematic Metabolic Engineering for Enhanced Spinosad Biosynthesis in *Saccharopolyspora spinosa* NHF132**

*Shuliu Wang^1^, Yuxin Liu^1^, Qian Zhang^1^, Yue Jiang^1^, Xiaoqian Zeng^1^, Chengyu Zhang^1^, Jiagao Cheng^1,2^, Weishan Wang^3^, Lixin Zhang^1,^ *, Gao-Yi Tan^1,^ **

^1^ State Key Laboratory of Bioreactor Engineering (SKLBE), and School of Biotechnology, East China University of Science and Technology (ECUST), Shanghai 200237, China

^2^ Shanghai Key Laboratory of Chemical Biology, School of Pharmacy, East China University of Science and Technology, Shanghai 200237, China

^3^ State Key Laboratory of Microbial Resources and CAS Key Laboratory of Pathogenic Microbiology and Immunology, Institute of Microbiology, Chinese Academy of Sciences (CAS), Beijing 100101, China

* To whom correspondence may be addressed. Email: [lxzhang@ecust.edu.cn](mailto:lxzhang@ecust.edu.cn); [tangy@ecust.edu.cn](mailto:tangy@ecust.edu.cn)

**Table S1. Strains used in this work**

| ***Saccharopolyspora spinosa* Strain** | **Genotype** | **Reference** |
| --- | --- | --- |
| NHF132 | Wild-type strain | Laboratory stock |
| NHF132-BAC | NHF132:: *spn*-BGC | This work |
| NHF132-Rham-GHIK | NHF132:: *kasop**-Rham, *spnGHIK* | This work |
| NHF132-*kasop**-TAG | NHF132:: *kasop**-*sco6196* | This work |
| NHF132-*cumate*-TAG | NHF132:: *cumate*-*sco6196* | This work |
| NHF132-*ermE**-*gusA* | NHF132:: *ermE**-*gusA* | This work |
| NHF132-*kasop**-*gusA* | NHF132:: *kasop**-*gusA* | This work |
| NHF132-*J23119*-*gusA* | NHF132:: *J23119*-*gusA* | This work |
| NHF132-*SP43*-*gusA* | NHF132:: *SP43-gusA* | This work |
| NHF132-*ermE**-NCM | NHF132:: *ermE*-*NCM | This work |
| NHF132-*kasop**-NCM | NHF132:: kasop*-NCM | This work |
| NHF132-*SP43*-NCM | NHF132:: *SP43*-NCM | This work |
| NHF132-ΔEry | NHF132:: ΔEry | This work |
| NHF132-ΔFla | NHF132:: ΔFla | This work |
| NHF132-ΔGes | NHF132:: ΔGes | This work |
| NHF132-TAG-NCM | NHF132:: *cumate-sco6196*, *SP43*-NCM | This work |
| NHF132-BAC-*ermE**-NCM | NHF132:: *spn*-BGC, *kasop**-Rham, *ermE**-NCM | This work |
| NHF132-BAC-*kasop**-NCM | NHF132:: *spn*-BGC, *kasop**-Rham, *kasop**-NCM | This work |
| NHF132-BAC-*SP43*-NCM | NHF132:: *spn*-BGC, *kasop**-Rham, *SP43*-NCM | This work |
| ***Escherichia coli* Strain** | **Genotype** | **Reference** |
| DH10b | *F- mcrA Δ(mrr-hsdRMS-mcrBC)* Φ*80lacZΔM15 ΔlacX74 recA1 endA1 araD139 Δ(ara, leu)7697 galE15 galK λ-rpsL nupG* | Laboratory stock |
| S17-1 | RP4 derivative integrated in chromosome | Laboratory stock |

**Table S2. Plasmids used in this work**

| **Plasmid** | **Relevant characteristics** | **Reference** |
| --- | --- | --- |
| pSET152 | Integrative *E. coli-Streptomyces* shuttle vector, attP (ΦC31 integrase), Apr^R^ | Laboratory stock^[1]^ |
| pBBR-*gusA* | pBBR carrying gusA (without promoter), Kan^R^ | Laboratory stock^[2]^ |
| pSET152-*gusA* | Derived from pSET152, Apr^R^ | This work |
| pSET152-*kasop*-gusA* | Derived from pSET152-*gusA*, expression driven by *kasop** promoter, Apr^R^ | This work |
| pSET152-*ermE*-gusA* | Derived from pSET152-*gusA,* expression driven by *ermE** promoter, Apr^R^ | This work |
| pSET152-*J23119-gusA* | Derived from pSET152-*gusA*, expression driven by *J23119* promoter, Apr^R^ | This work |
| pSET152-*SP43-gusA* | Derived from pSET152-*gusA,* expression driven by *SP43* promoter, Apr^R^ | This work |
| pRham-GHIK | Derived from pSET152, expressing Rham and *spnGHIK*, Apr^R^ | This work |
| pRham-Amp | Derived from pRham-GHIK, encoding an extra Amp^R^, Apr^R^, Amp^R^ | This work |
| pCu-SCO6196 | Derived from pSET152, expressing the cumate inducible *sco6196*, Apr^R^ | Laboratory stock^[3]^ |
| pKas-SCO6196 | Derived from pSET152, expressing the *kasop**- controlled *sco6196*, Apr^R^ | Laboratory stock^[3]^ |
| pCu-TAG | Derived from pSET152, expressing the cumate inducible *sco6196*, Apr^R^ | This work |
| pKas-TAG | Derived from pSET152, expressing the *kasop**- controlled *sco6196*, Apr^R^ | This work |
| pSET152-*ermE*-BauA-ermE*-MCR-C* | Derived from pSET152, expressing *BauA* and *MCR-C*, Apr^R^ | Gift from Zaigao Tan^[4]^ |
| pSET152-*kasop**-*BauA-SPL42-MCR-C* | Derived from pSET152, expressing *BauA* and *MCR-C*, Apr^R^ | Gift from Zaigao Tan^[4]^ |
| pSET152-*J23119-BauA-J23119-MCR-C* | Derived from pSET152-*kasop*-BauA-SPL42-MCR-C*, expression driven by *J23119* promoter, Apr^R^ | This work |
| pSET152-*SP43-BauA-SP43-MCR-C* | Derived from pSET152-k*asop*-BauA-SPL42-MCR-C,* expression driven by *SP43* promoter, Apr^R^ | This work |
| pBAC2015-81kb-J1074 | Derived from pBAC2015-CS81, containing the whole spinosad biosynthetic gene cluster (81kb), Chl^R^ | Laboratory stock^[5]^ |
| pBAC-Spn | Derived from pBAC2015-81kb-J1074, Apr^R^ | This work |
| pSC101 | *In vivo* editing plasmid, encoding the arabinose-inducible ETγA operon, Tet^R^ | Laboratory stock^[6]^ |
| pBAC-Rham-(*ermE**)-NCM | Derived from pBAC-Spn*,* expressing *BauA* and *MCR-C*, expression driven by *ermE** promoter, Apr^R^, Amp^R^ | This work |

**Table S2. Plasmids used in this work (continued)**

| **Plasmid** | **Relevant characteristics** | **Reference** |
| --- | --- | --- |
| pBAC-Rham-(*kasop**)-NCM | Derived from pBAC-Spn*,* expressing *BauA* and *MCR-C*, expression driven by *kasop** promoter, Apr^R^, Amp^R^ | This work |
| pBAC-Rham-(*SP43*)-NCM | Derived from pBAC-Spn*,* expressing *BauA* and *MCR-C*, expression driven by*SP43* promoter, Apr^R^, Amp^R^ | This work |
| pTAG-NCM | Derived from pSET152-*SP43-BauA-SP43-MCR-C*, expressing BauA and MCR-C, expression driven by *SP43* promoter, Apr^R^, Amp^R^ | This work |
| pWT297 | A *Sa. spinosa-E. coli* shuttle replicative vector contains *Sa. spinosa* origin and *E. coli* origin pBR322, Apr^R^ | Gift from Yinhua Lu^[7]^ |
| pWT297-Ery-HR | Derived from pWT297, expressing crRNA and HR for *Ery* knockout, Apr^R^ | This work |
| pWT297-Fla-HR | Derived from pWT297, expressing crRNA and HR for *Fla* knockout, Apr^R^ | This work |
| pWT297-Ges-HR | Derived from pWT297, expressing crRNA and HR for *Ges* knockout, Apr^R^ | This work |

**Table S3. Primers used for plasmid/strain construction**

| **Plasmid** | **Primer Name** | **Sequence** |
| --- | --- | --- |
| **Plasmid construction** | | |
| pSET152-*gusA* | gusA-F | gggctgcaggtcgactctagatgttacgtcctgtagaaacccc |
|  | gusA-R | attcgatatcgcgcgcggccaccgatacaattaaaggctccttt |
| pSET152-*ermE**-*gusA* | ermE-F | gcggtcgatcttgacggctg |
|  | gusA-R-E-R | gacgtaacatttccgtccgtacctccgttg |
| pSET152-*kasop**-*gusA* | kasop-F | ggaacgatcgttggctgtgt |
|  | gusA-R-K-R | gacgtaacatatggcgtatcccctttcag |
| pSET152-*J23119*-*gusA* | J23119-F | ttgacagctagctcagtcct |
|  | gusA-R-J-R | gacgtaacatgcgtatcccctttcagatacgct |
| pSET152-*SP43*-*gusA* | SP43-F | tgttcacattcgaaccgtct |
|  | gusA-R-SP-R | caggacgtaacatgcgtatcccctttcagatac |
| pRham-GHIK | gtt-HR-F | tctgaaaggggatacgccatatgaaggggatcgtgctgg |
|  | gtt-HR-R | cccctttcagataccaattctcagccccgcgcgccgga |
|  | epi-HR-F | gtatctgaaaggggatacgcatgcaggtacgtcgacttgaca |
|  | epi-HR-R | tgtgaacacagccacaattctcagccggggctgcgcag |
|  | gdh-HR-F | tctgaaaggggatacgccatatgcggattctggtcaccgg |
|  | kre-HR-R | atgattacgaattcgatatctcagcgggttcggagggt |
|  | spnGHI-HR-F | ctaactcacattaattgcgttctggaaccgagcaggaaacac |
|  | spnGHI-HR-R | atcgtgatgagctcccgcccctccttttctgactgg |
|  | spnK-HR-F | cagaaaaggaggggcgggagctcatcacgatgtccacaac |
|  | spnK-HR-R | cttttgctgatggagctgtcactcgtcctccgcgctg |
| pRham-Amp | Amp-152-HR-F | agatccttttggttcatgtgcgcggaacccctatttgtttat |
|  | Amp-Rham-HR-R | agcctgaatggcgaatggcgttaccaatgcttaatcagtgaggcac |
| pCu-TAG | Cu-LacZa-HR-F | ggctgcaggtcgactctagattatcaccgcttgaacttggcg |
|  | sco6196-HR-R | cgatatcgtaatgcttatcatcaggggcgcgctccgta |
| pKas-TAG | kasop*-LacZa-HR-F | ggctgcaggtcgactctagatgttcacattcgaacggtctctg |
|  | sco6196-HR-R | cgatatcgtaatgcttatcatcaggggcgcgctccgta |
| pSET152-*J23119-BauA-J23119-MCR-C* | Lac-J23119-F | ttgggctgcaggtcgactctagattgacagctagctcagtcctaggtataatgctagcgtatctgaaaggggatacgcatgaatcagccgctgaacgtgg |
|  | BauA-J23119-R | gctagcattatacctaggactgagctagctgtcaatctagagtcgacctgcagcccaa |
|  | BauA-J23119-F | atggcagatctcagcgccac |
|  | MCR-J23119-R | gtggcgctgagatctgccatgcgtatcccctttcagatacgctagcattatacctaggactgagctagctgtcaattacgcaatgccgttcagcgcttc |

**Table S3. Primers used for plasmid/strain construction (continued)**

| **Plasmid** | **Primer Name** | **Sequence** |
| --- | --- | --- |
| **Plasmid construction** | | |
| pSET152-*SP43*-*BauA*-*SP43*-*MCR-C* | Lac-SP43-F | ttgggctgcaggtcgactctagatgttcacattcgaaccgtctctgc |
|  | BauA-SP43-R | ccacgttcagcggctgattcatgcgtatcccctttcagatactggc |
|  | BauA-SP43-F | gaagcgctgaacggcattgcgtaatgttcacattcgaaccgtctctgc |
|  | MCR-SP43-R | gtggcgctgagatctgccatgcgtatcccctttcagatactggc |
| pTAG-NCM | TAG-NCM-HR-F | cgctcggtcgttcggctagattatcaccgcttgaacttggcg |
|  | TAG-NCM-HR-R | gctgataccgctcgccgcaggatatcgtaatgcttatcatcaggggc |
|  | pNCM-F | ctgcggcgagcggtatca |
|  | pNCM-R | tctagccgaacgaccgagc |
| pBAC-Rham-(*ermE**)-NCM | Rham-NCM-HR-F | catgtcatagctgtttcctgcgcggaacccctatttgttt |
|  | Rham-BAC-HR-R | ccgctcgtggccatcggcctacgcaattaatgtgagttagctcac |
|  | ermE-NCM-BAC-HR-F | ctatgaccatgattacgccagcgagtgtccgttcgagt |
|  | NCM-Rham-HR-R | aaacaaataggggttccgcgcaggaaacagctatgacatgattacga |
| pBAC-Rham-(*kasop**)-NCM | Rham-NCM-HR-F | *See above* |
|  | Rham-BAC-HR-R | *See above* |
|  | kasop*-NCM-BAC-HR-F | ctatgaccatgattacgccaggaacgatcgttggctgtgt |
|  | NCM-Rham-HR-R | *See above* |
| pBAC-Rham-(*SP43*)-NCM | Rham-NCM-HR-F | *See above* |
|  | Rham-BAC-HR-R | *See above* |
|  | SP43-NCM-BAC-HR-F | ctatgaccatgattacgccatgttcacattcgaaccgtctctg |
|  | NCM-Rham-HR-R | *See above* |
| pWT297-Ery-HR | Ery-UHR-F | ttattggtgagaattcatcgatgatgtgttggtggtgccgaatcg |
|  | Ery-UHR-R | gtcacggcggtttccactaacggtgcggaccctacttgattgcttcg |
|  | Ery-DHR-F | acgcgaagcaatcaagtagggtccgcaccgttagtggaaaccgcc |
|  | Ery-DHR-R | ctatagggagaccggcagatctgatcgatcgtcccatcggaccat |

**Table S3. Primers used for plasmid/strain construction (continued)**

| **Plasmid** | **Primer Name** | **Sequence** |
| --- | --- | --- |
| **Plasmid construction** | | |
| pWT297-Fla-HR | Fla-UHR-F | ttattggtgagaattcatcgatgattgcgactgcggatgctgtcg |
|  | Fla-UHR-R | cgccggaggatggcaggattccgcccggcgtgtcttgcgttgtgg |
|  | Fla-DHR-F | aagcaccacaacgcaagacacgccgggcggaatcctgccatcctc |
|  | Fla-DHR-R | ctatagggagaccggcagatctgattcatgactccgggcagagcc |
| pWT297-Ges-HR | Ges-UHR-F | ttattggtgagaattcatcgatgatgttcgtgttctgcttccggt |
|  | Ges-UHR-R | ccccggcgctggaagaagccgtcgcaaggcggaacccgggctcat |
|  | Ges-DHR-F | cgcggatgagcccgggttccgccttgcgacggcttcttccagcgc |
|  | Ges-DHR-R | actcactatagggagaccggcagatctgatcgagttcagctcagcacgtt |

**Table S3. Primers used for plasmid/strain construction (continued)**

| **Plasmid/Strain** | **Primer Name** | **Sequence** |
| --- | --- | --- |
| **PCR verification** | | |
| pRham-GHIK | Int-v-F | tccgatggcgcgagtgcca |
|  | Epi-v-R | gcagaggtaggtcatcacgg |
|  | SpnK-v-F | tgtccgttgtggacgatcag |
|  | ori-v-R | aagaactctgtagcaccgcc |
| NHF132-Rham-GHIK | 132-up-v-F | atggcgttgtaaacgcttcg |
|  | 132-up-v-R | caggacatatccacgccctc |
|  | Kre-v-F | tgtccgttgtggacgatcag |
|  | SpnG-v-R | gccatgagatgcgtcggata |
|  | 132-dn-v-F | cgacttcgggagtggtcatc |
|  | 132-dn-v-R | tgatcttctccggcacttgg |
| pBAC-Spn | Apr-v-F | ccgagacactgcaccattct |
|  | traJ-v-R | aacgaagagcgattgaggaa |
|  | BAC-Chl-v-F | ccgaagcacagtccctactc |
|  | BAC-Chl-v-R | aagatgtggcgtgttacggt |
| NHF132-BAC-Spn | 132-up-v-F | *See above* |
|  | SpnE-v-R | gtggacagatgactcctggc |
|  | BAC-Chl-v-F | *See above* |
|  | BAC-Chl-v-R | *See above* |
| pCu-TAG | Int-v-F | *See above* |
|  | Amp-v-R | ctcaacagcggtaagatcct |
| NHF132-*cumate*-TAG | Amp-v-F | acgatcaaggcgagttacat |
|  | Sco6196-v-R | ttcaccatgatgacgccgat |
| pGusA | Int-v-F | *See above* |
|  | gusA-v-R | tcttgccgttttcgtcggta |
| pNCM-(*ermE*/kasop*/J23119/SP43*) | Int-v-F | *See above* |
|  | MCR-C-v-R | attggcgaagagggtatggc |
| NHF132-*ermE*/kasop*/J23119/SP43*-NCM | 132-up-v-F | *See above* |
|  | MCR-C-v-R | *See above* |
| pWT297-Ery-HR | Ery-UHR-v-F | cacacaccagaaggaagcct |
|  | Ery-UHR-v-R | cgagtaggccagcttctcag |
|  | Ery-DHR-v-F | ggcttgtgtctcgtctgtga |
|  | Ery-DHR-v-R | tctctttcggttacggtgcc |
| pWT297-Fla-HR | Fla-UHR-v-F | ccaagtctgtccatggcctt |
|  | Fla-UHR-v-R | acctcacccgaaaacccatc |
|  | Fla-DHR-v-F | aaacgcgaggtcagctacaa |
|  | Fla-DHR-v-R | gtcaccgactacgccagaaa |
| pWT297-Ges-HR | Ges-UHR-v-F | acgatcagctctttgtccgg |
|  | Ges-UHR-v-R | gagctgtgtgtggaagagct |
|  | Ges-DHR-v-F | gagagcttcgtcggatccag |
|  | Ges-DHR-v-R | ctcggaacagatcgccaagt |

**Table S3. Primers used for plasmid/strain construction (continued)**

| **Plasmid/Strain** | **Primer Name** | **Sequence** |
| --- | --- | --- |
| **PCR verification** | | |
| NHF132-ΔEry | 132-Ery-up-v-F | cgtccggtgggagtgtttta |
|  | 132-Ery-dn-v-R | ggagttctttcccgacagca |
| NHF132-ΔFla | 132-Fla-up-v-F | gccgatagcaccgagatcaa |
|  | 132-Fla-dn-v-R | catcagcagatcccgcagat |
| NHF132-ΔGes | 132-Ges-up-v-F | cgcggcgaagaatattgtgg |
|  | 132-Ges-dn-v-R | gatgggcaggttcacgaaga |
| pTAG-NCM | Int-v-F | *See above* |
|  | MCR-C-v-R | *See above* |
|  | MCR-C-v-F | cgggcgtaaagacagtgact |
|  | Amp-v-R | *See above* |
|  | sco6196-v-F | ccaccacaacatcctcaa |
|  | ori-v-R | *See above* |
| NHF132-TAG-NCM | 132-up-v-F | *See above* |
|  | MCR-C-v-R | *See above* |
|  | MCR-C-v-F | *See above* |
|  | Amp-v-R | *See above* |
| pBAC-Rham-(*ermE*/kasop*/SP43*)-NCM | BAC-v-F | tgaccaatggttccccgaag |
|  | BauA-v-R | ggtatgcggcagatgatcca |
|  | MCR-C-v-F | *See above* |
|  | Rham-v-R | caacgatcgttccggatcct |
|  | Rham-v-F | ttacgtgttccccggtgatg |
|  | BAC-v-R | cgatgcctggattcattgcg |
| NHF132-BAC-(*ermE*/kasop*/SP43*)-NCM | 132-up-v-F | *See above* |
|  | MCR-C-v-R | *See above* |
|  | Kre-v-F | *See above* |
|  | BAC-v-R | *See above* |

**Table S4. Primers used for qPCR**

| **Target** | **Primer Name** | **Sequence** |
| --- | --- | --- |
| Int | phiC31-qPCR-F | tggtggcgtgagatcaagac |
|  | phiC31-qPCR-R | agcggtcttcttcccaatcg |
| Internal reference | rpL13-qPCR-F | acttctcgaccttcgcgttg |
|  | rpL13-qPCR-R | gctcgaaaaggcgatcaagg |
|  | sigA-qPCR-F | gcacatggtcgaggtgatca |
|  | sigA-qPCR-R | gatctccagcaccttctccg |
| TAG | TAG-qPCR-F | ggtgatggggaatctgggtg |
|  | TAG-qPCR-R | ttcagctccgcgatgaacat |
| NCM pathway | BauA-qPCR-F | gcgccgcattttgaaaaagg |
|  | BauA-qPCR-R | tttcatgcccgcttcaaacg |
|  | MCR-C-qPCR-F | caggctatcactcgctacgg |
|  | MCR-C-qPCR-R | gttcgcacaactcggcaaat |
| Spn-PKS | spnE-qPCR-F1 | cgaaatacgccgaccattcg |
|  | spnE-qPCR-R1 | gccctgattcccgattgtca |
|  | spnE-qPCR-F2 | cgcagtagaccacgacatca |
|  | spnE-qPCR-R2 | cctgcttggtgatactgcct |
|  | SpnD-qPCR-F1 | agcatcagtggtgttctccg |
|  | SpnD-qPCR-R1 | ggatcgtgacgagacatccc |
|  | SpnD-qPCR-F2 | ggtcaccgcaagcgaataac |
|  | SpnD-qPCR-R2 | gttctgtccgcgattttccg |
|  | SpnC-qPCR-F1 | tgagacaagactcgttcggc |
|  | SpnC-qPCR-R1 | aagctacgaatgcgccagat |
|  | SpnC-qPCR-F2 | accctggtgtgctcatttcc |
|  | SpnC-qPCR-R2 | tttcattcgcaccggatgga |
|  | SpnB-qPCR-F1 | tcgaacaactccaccagctc |
|  | SpnB-qPCR-R1 | ggagttggggaagacggatg |
|  | SpnB-qPCR-F2 | cgtcgggtagttgaacacca |
|  | SpnB-qPCR-R2 | gccggttctcaggagatcac |
|  | SpnA-qPCR-F1 | actcaccatcgccacatgtt |
|  | SpnA-qPCR-R1 | ttggtacgccacactgtctc |
|  | SpnA-qPCR-F2 | ctgaatccaccgtcaggctt |
|  | SpnA-qPCR-R2 | gatctctgacgactacgccg |
| Rhamnose | gtt-qPCR-F | ggtgtacgacaagccgatga |
|  | gtt-qPCR-R | tcgatccgaatgccgaactg |
|  | epi-qPCR-F | acttgacatcacgggtgcat |
|  | epi-qPCR-R | gagacgctgtggttggtctg |
|  | gdh-qPCR-F | atcacgaacctgatggacgg |
|  | gdh-qPCR-R | atgttgtagatctcgcccgc |
|  | kre-qPCR-F | aaacgatgatccgcctctcg |
|  | kre-qPCR-R | cttctgctccggtccacg |

**Table S4. Primers used for qPCR (continued)**

| **Target** | **Primer Name** | **Sequence** |
| --- | --- | --- |
| Rhamnose-related | SpnG-qPCR-F | caccgacctacccgacaac |
|  | SpnG-qPCR-R | tactggggaagcacgagttg |
|  | SpnH-qPCR-F | tcgacgaacaggtcaggttc |
|  | SpnH-qPCR-R | gagcttcgggtacaggttcc |
|  | SpnI-qPCR-F | atgtccagcccgtaaacgag |
|  | SpnI-qPCR-R | ctacgagcaccacttctccc |
|  | SpnK-qPCR-F | tcagcctgctcaagaacctg |
|  | SpnK-qPCR-R | tgacgcctttctccaggaac |
| Spn-BGC | SpnJ-qPCR-F | ctaccttcaaggcgtcgagg |
|  | SpnJ-qPCR-R | tggtgacgatcccgaagttg |
|  | SpnP-qPCR-F | cactttgctccccgtactgt |
|  | SpnP-qPCR-R | catctcgatgcgtttcgtgc |
|  | SpnS-qPCR-F | ccgaagcacagtccctactc |
|  | SpnS-qPCR-R | ggtacctcagggactcgtct |

**Table S5. Promotor gene sequences used in this work**

| **Name** | **Sequence** |
| --- | --- |
| *ermE** | gcggtcgatcttgacggctggcgagaggtgcggggaggatctgaccgacgcggtccacacgtggcaccgcgatgctgttgtgggcacaatcgtgccggttggtaggatc |
| *kasop** | ggaacgatcgttggctgtgttcacattcgaaccgtctctgctttgacaacatgctgtgcggtgttgtaaagtcgtggccaggagaatacgacaggtatctgaaaggggatacgc |
| *J23119* | ttgacagctagctcagtcctaggtataatgctagc |
| *SP43* | tgttcacattcgaaccgtctctgctttgacacggacaagcgctatggtgtaaagtcgtggcca |

**Table S6. HPLC detection conditions for spinosad (spinosyn A/D)**

| **Time** | **A %** | **B %** | **ml/min** | **Pressure bar** |
| --- | --- | --- | --- | --- |
| 3.00 | 50.0 | 50.0 | 0.600 | 400.00 |
| 15.00 | 80.0 | 20.1 | 0.600 | 400.00 |
| 17.00 | 99.0 | 1.0 | 0.600 | 400.00 |
| 20.00 | 99.0 | 1.0 | 1.000 | 400.00 |
| 20.10 | 50.0 | 50.0 | 1.000 | 400.00 |
| 25.00 | 50.0 | 50.0 | 1.000 | 400.00 |

**Table S7. All dataset of the flux analysis of Spn-GEM (in separate Excel)**

**Table S8. All reaction scores of Spn-GEM (in separate Excel)**

**Table S9. Spn-GEM prediction targets. (in separate Excel)**

**Table S10. Antismash predicted BGCs in *Sa. spinosa* NHF132 (in separate Excel)**

**Table S11. Original qPCR data (in separate Excel)**


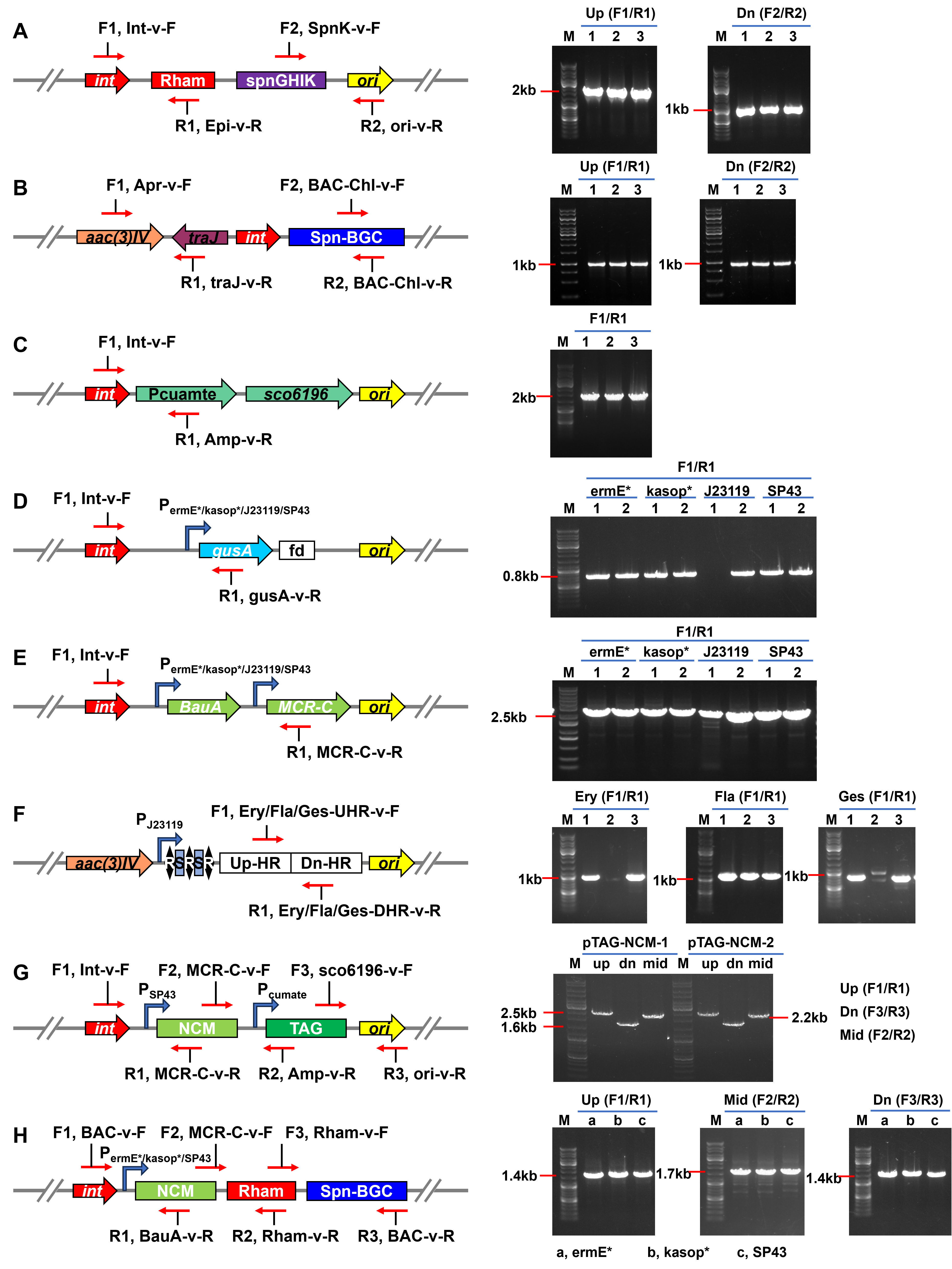


**Figure S1. Agarose-gel electrophoresis verification of plasmid construction.** (A-H) PCR verification results for plasmid construction: A, pRham-GHIK; B, pBAC-Spn; C, pCu-TAG; D, pGusA; E, pNCM; F, pWT297-BGC-HR; G, pTAG-NCM; H, pBAC-Rham-NCM. Numbers 1, 2, and 3 represent three random transformants. The primer sequences used for PCR verification are provided in the “PCR verification” section of Table S3.


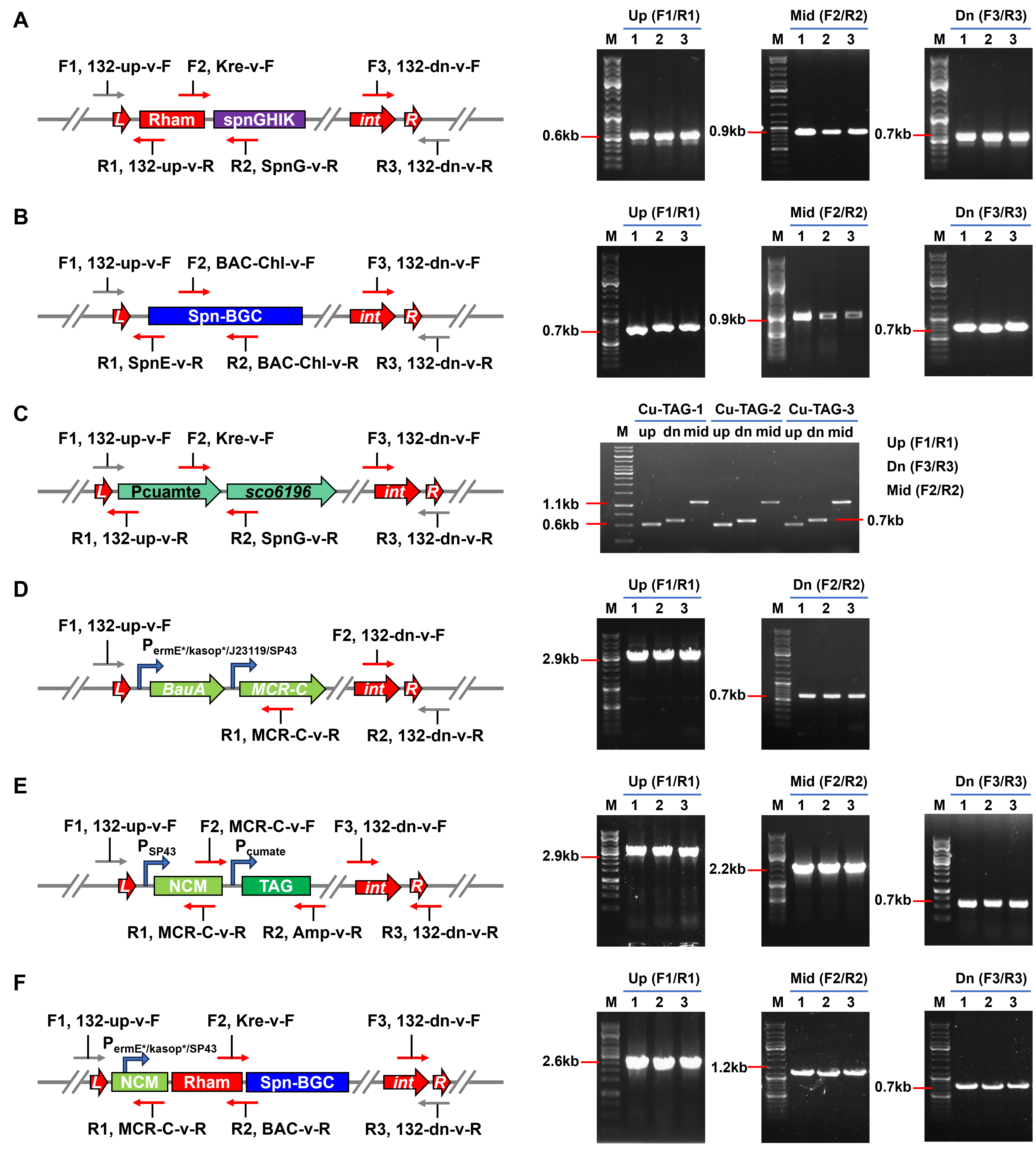


**Figure S2. Agarose-gel electrophoresis verification of engineered strains construction.** (A-F) PCR verification results of engineered strains construction: A, NHF132-Rham-GHIK; B, NHF132-BAC-Spn; C, NHF132-*cumate*-TAG; D, NHF132-(*ermE*/kasop*/J23119/SP43*)-NCM; E, NHF132-TAG-NCM; F, NHF132-BAC-(*ermE*/kasop*/SP43*)-NCM. Numbers 1, 2, and 3 represent three random transformants. The primer sequences used for PCR verification are provided in the “PCR verification” section of Table S3.


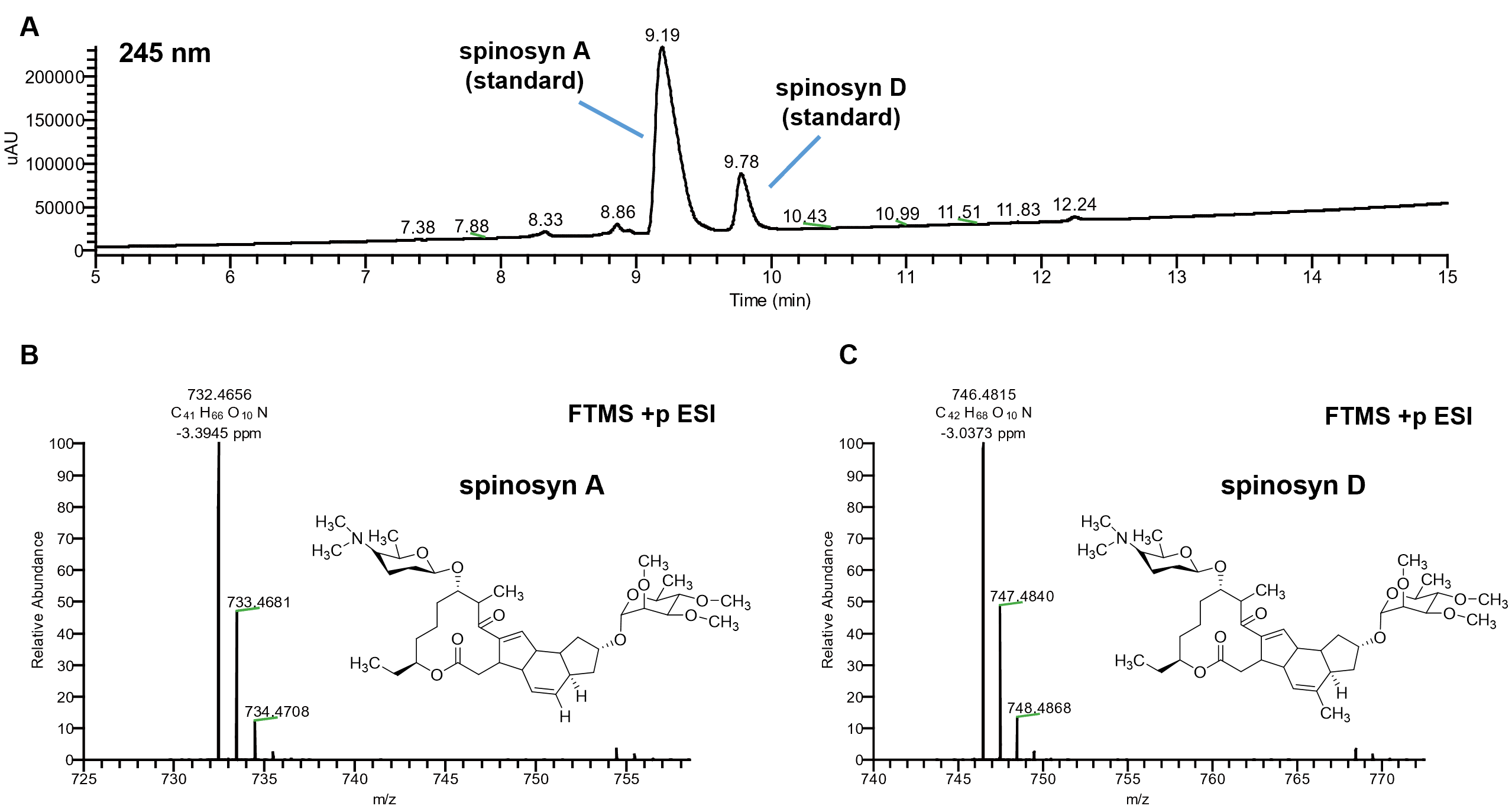


**Figure S3. The QExactive (UPLC-MS) analysis of spinosad A and D.** (A) HPLC profiles of spinosad A and D monitoring with Photodiode Array Detector (PDA) at 245 nm. (B) High-resolution MS spectra of spinosad A in positive-ion mode ([M+H]^+^). (C) High-resolution MS spectra of spinosad D in positive-ion mode ([M+H]^+^).


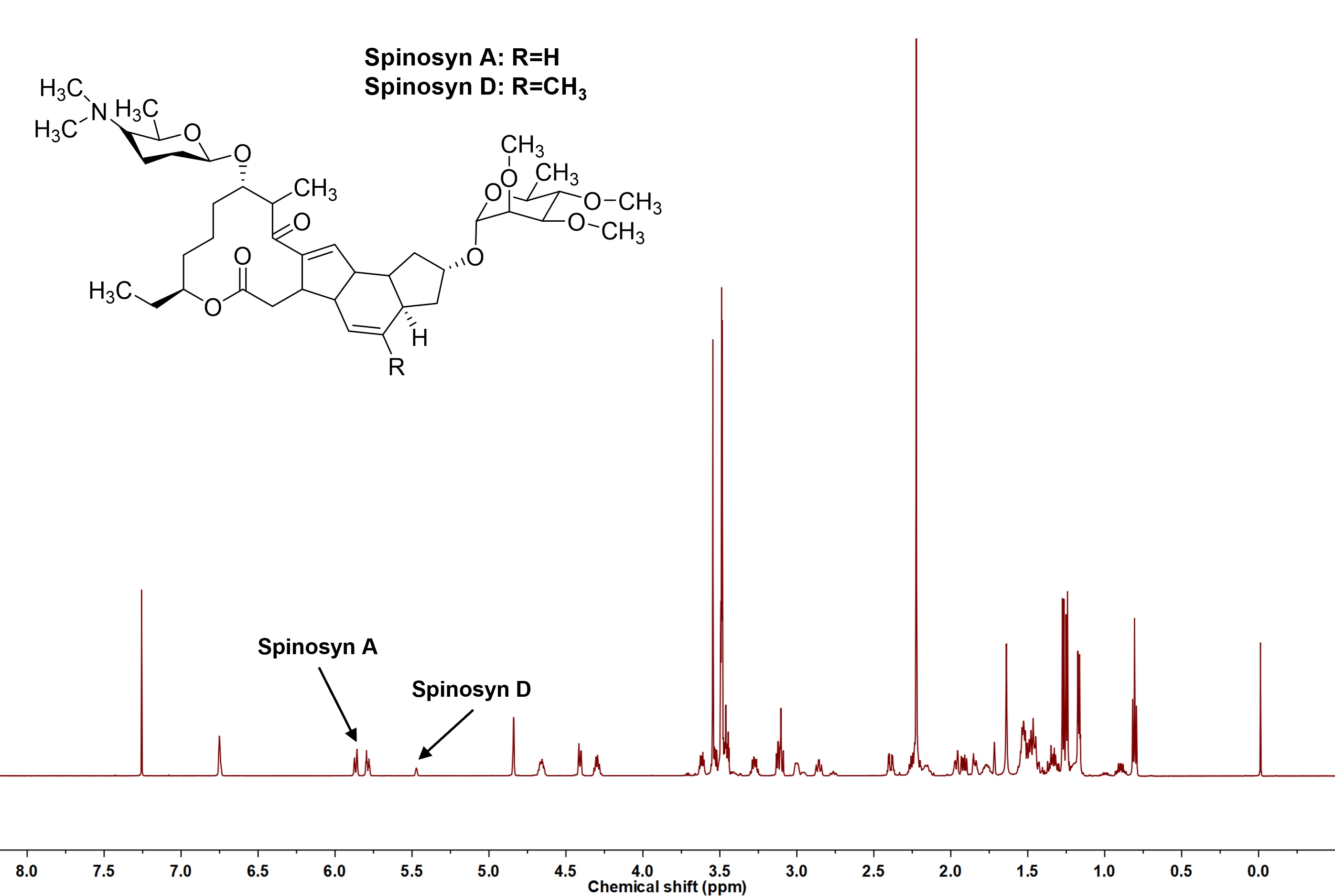


**Figure S4. ^1^H NMR spectra of spinosad (comprising ~82% spinosyn A and ~18% spinosyn D).** The spectra show well-resolved signals for spinosyn A (doublet at 5.86 ppm) and spinosyn D (singlet at 5.47 ppm), confirming the presence of both components. All samples were dissolved in deuterated chloroform (CDCl3) for analysis^[8]^.


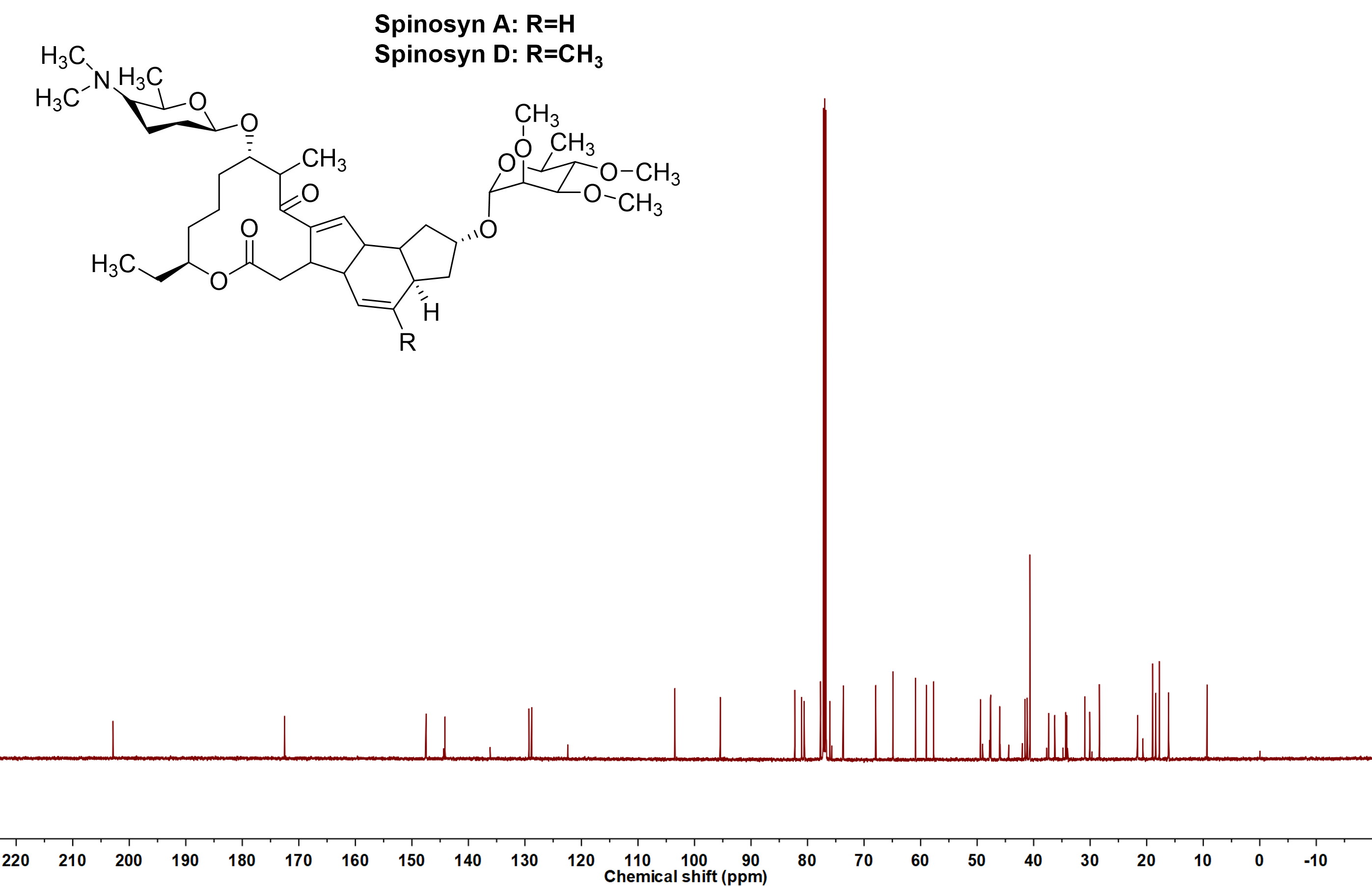


**Figure S5. ^13^C NMR spectra of spinosad (comprising ~82% spinosyn A and ~18% spinosyn D).** All samples were dissolved in deuterated chloroform (CDCl3) for analysis.


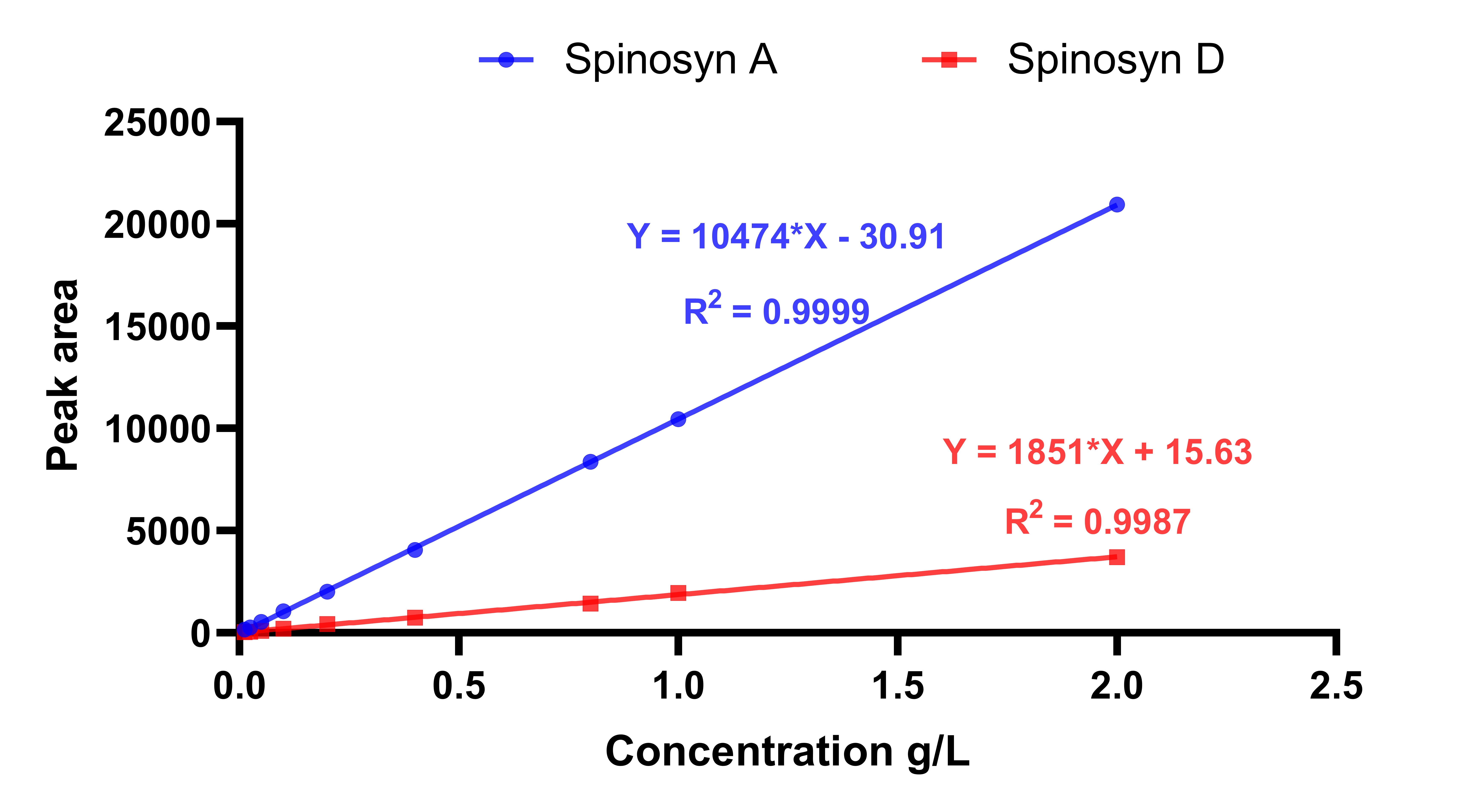


**Figure S6. The standard curve and the corresponding regression equation used for quantification of spinosad.** The standard spinosad (comprising ~82% spinosyn A and ~18% spinosyn D) was tested as a reference. The calibration curve was constructed by plotting the peak area against the known concentrations of the standards.


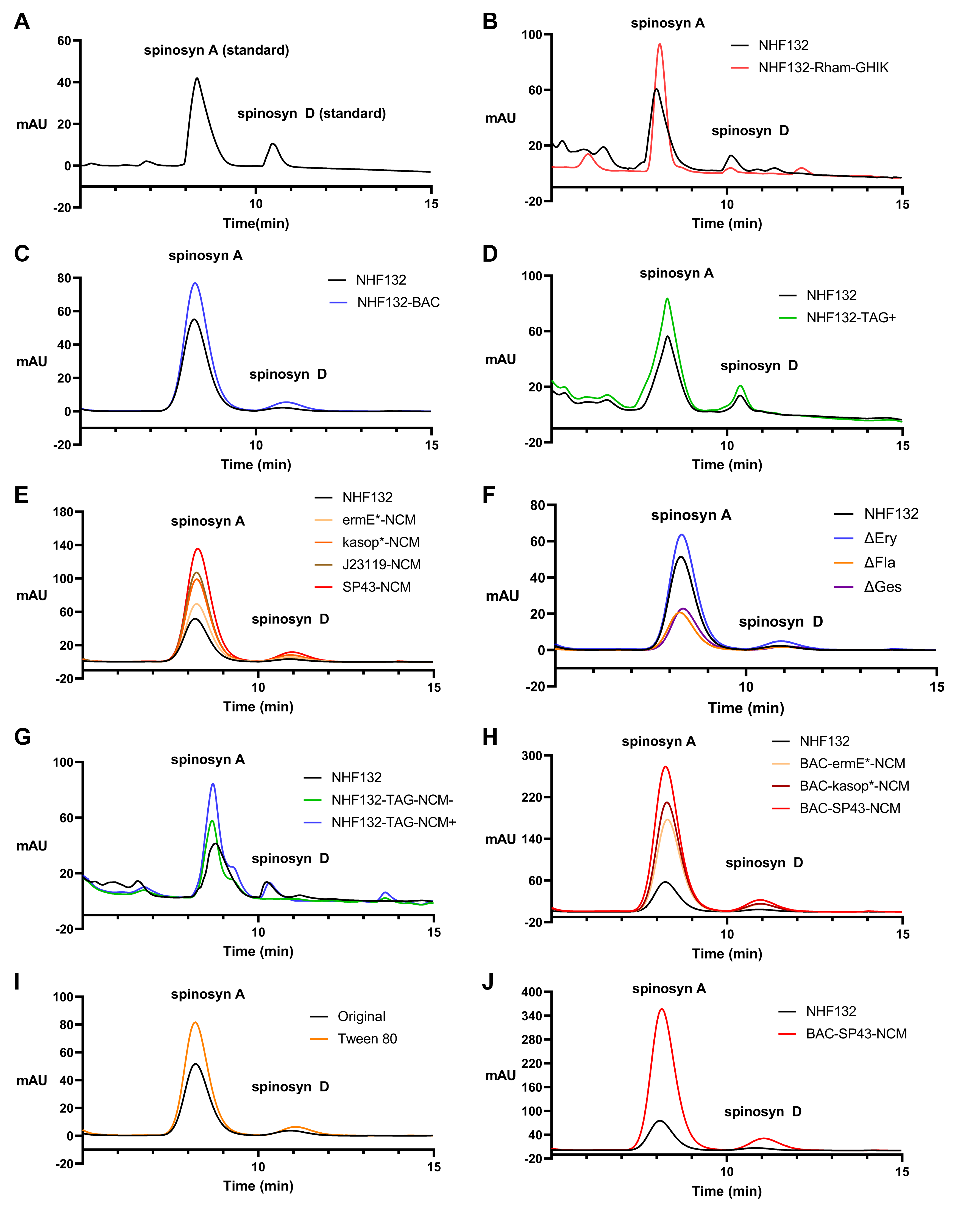


**Figure S7. Comparative HPLC chromatograms of the engineered and parental strains.** (A) Standard spinosad (comprising ~82% spinosyn A and ~18% spinosyn D). (B-J) Each subfigure presents the differences in spinosad production between the parental strain NHF132 and the engineered mutants under distinct engineering strategies: B, NHF132-Rham-GHIK; C, NHF132-BAC-Spn; D, NHF132-*cumate*-TAG^+^; E, NHF132-*ermE*/kasop*/J23119/SP43*-NCM; F, NHF132-ΔEry/ΔFla/ΔGes; G, NHF132-TAG-NCM; H, NHF132-BAC-(*ermE*/kasop*/SP43*)-NCM; I, NHF132 with and without 400 mg/L Tween 80 added; J, NHF132-BAC-*SP43*-NCM after medium and fermentation process optimization.


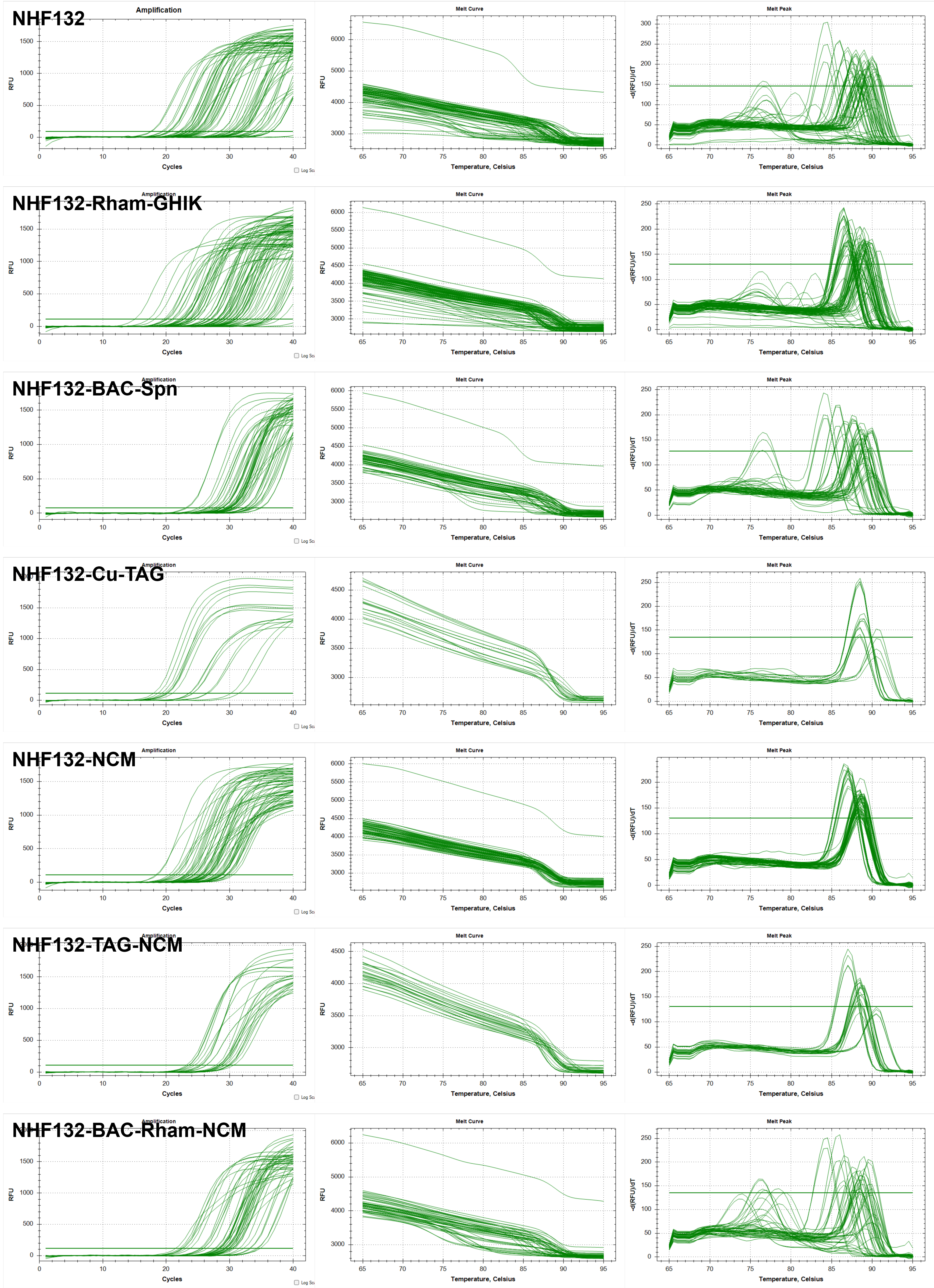


**Figure S8. qPCR original curves of the engineered and parental strains.** Each figure displays the amplification curves, melt curves, and melt peaks obtained during qPCR quantification of gene expression (primer sequences are provided in Table S4) in the parental strain NHF132 and its engineered mutants.


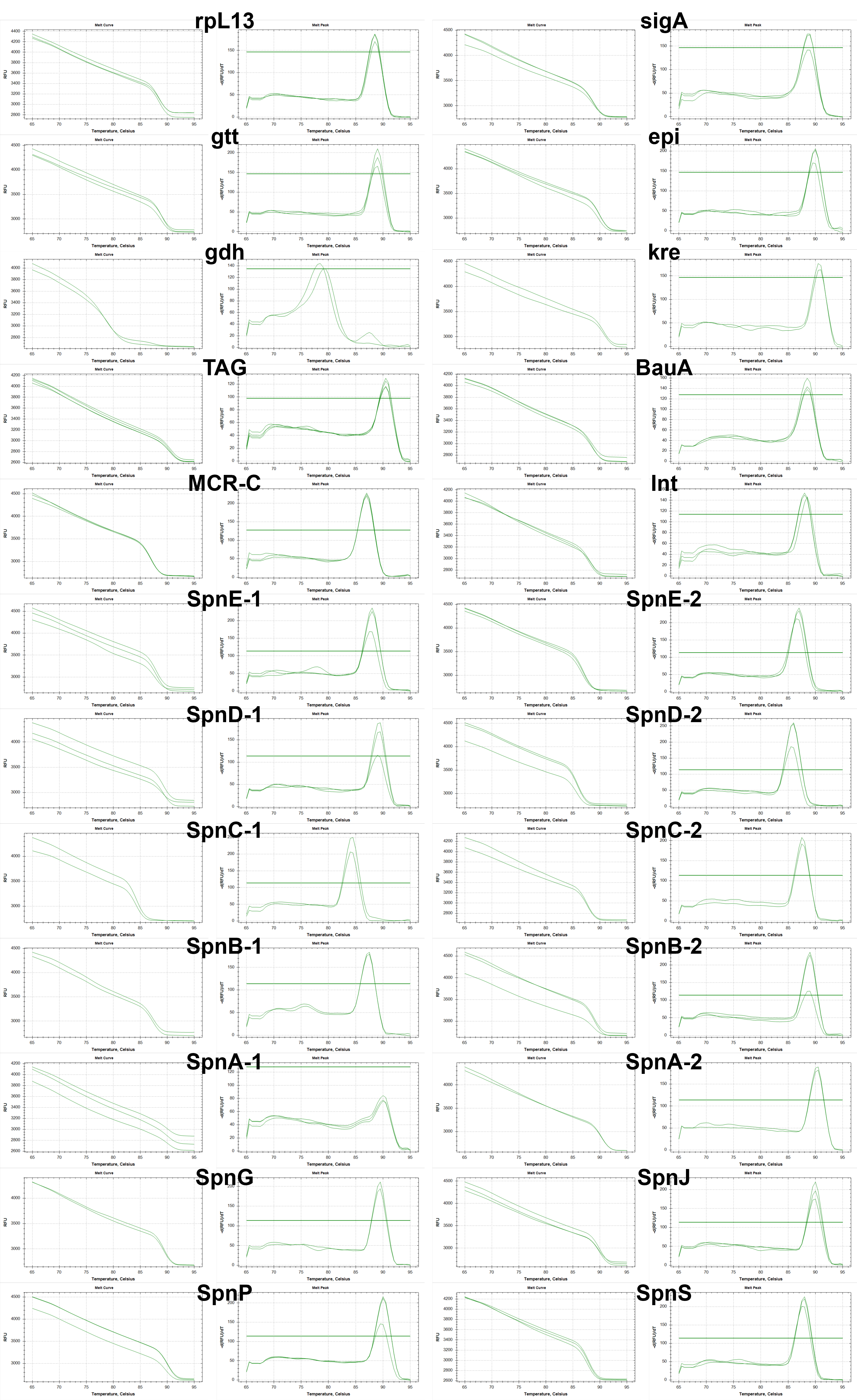


**Figure S9. qPCR original curves of** **each target genes.** Each figure displays the melt curves and melt peaks obtained during qPCR quantification of the indicated target genes, using the primer pairs listed in Table S4.


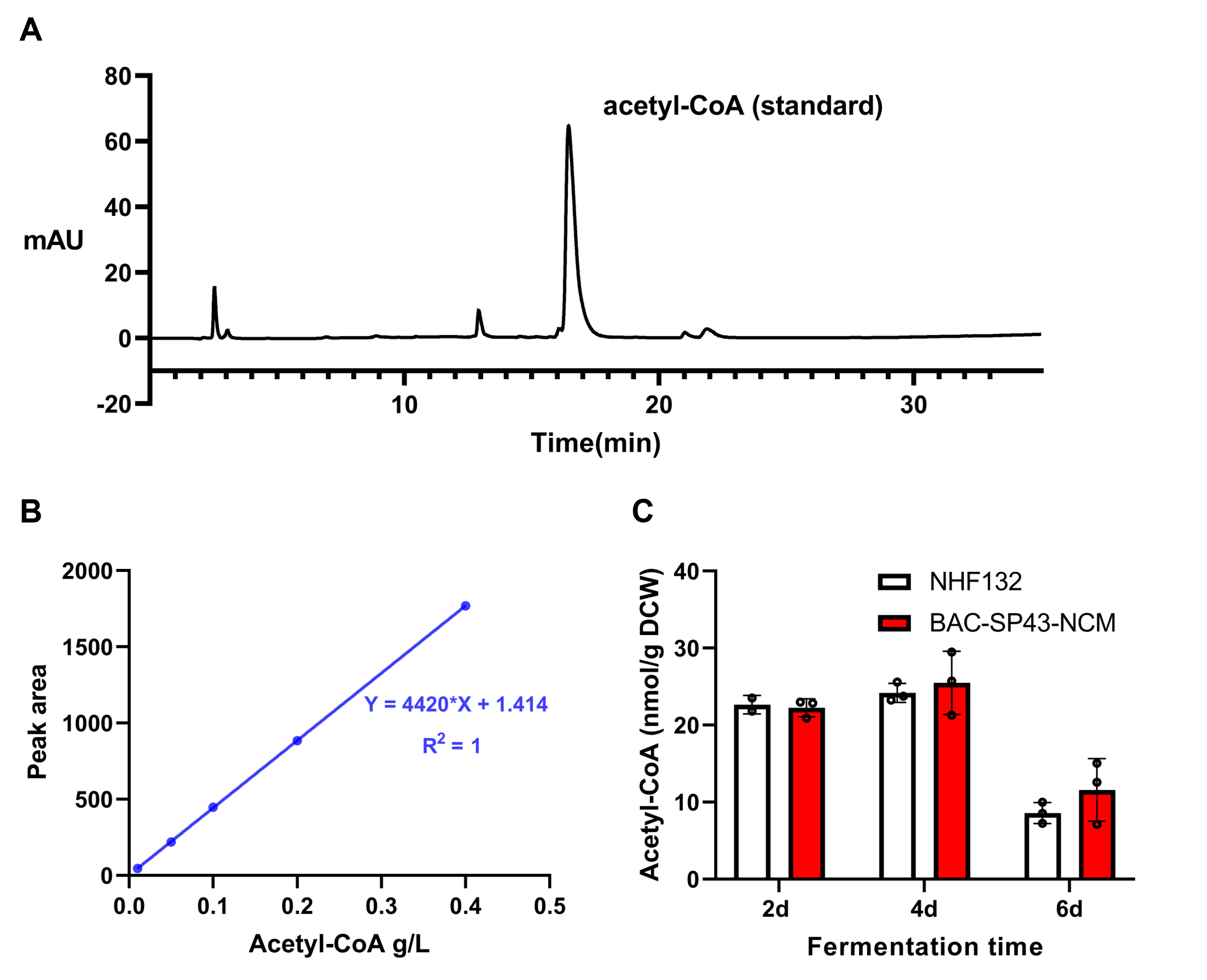


**Figure S10. Quantitative analysis of intracellular acetyl-CoA.** (A) HPLC chromatogram of acetyl-CoA standard. (B) The standard curve and the corresponding regression equation used for quantification of acetyl-CoA. (C) Intracellular acetyl-CoA level in wild-type strain NHF132 and engineered strain NHF132-BAC-*SP43*-NCM. Intracellular acetyl-CoA were extracted and quantified according to the method described with some modifications. ^[9,10]^

**References**

[1] F. Flett, V. Mersinias, C. P. Smith, "High efficiency intergeneric conjugal transfer of plasmid DNA from *Escherichia coli* to methyl DNA-restricting streptomycetes," *FEMS Microbiology Letters* (1997): 223. <https://doi.org/10.1111/j.1574-6968.1997.tb13882.x>.

[2] T. Shi, L. Zhang, M. D. Liang, et al., "Screening and engineering of high-activity promoter elements through transcriptomics and red fluorescent protein visualization in *Rhodobacter sphaeroides*," *Synthetic and Systems Biotechnology* (2021): 335. <https://doi.org/10.1016/j.synbio.2021.09.011>.

[3] W. Wang, S. Li, Z. Li, et al., "Harnessing the intracellular triacylglycerols for titer improvement of polyketides in *Streptomyces*," *Nature Biotechnology* (2020): 76. <https://doi.org/10.1038/s41587-019-0335-4>.

[4] J. Li, X. Mu, W. Dong, et al., "A non-carboxylative route for the efficient synthesis of central metabolite malonyl-CoA and its derived products," *Nature Catalysis* (2024): 361. <https://doi.org/10.1038/s41929-023-01103-2>.

[5] M. D. Liang, L. S. Liu, F. Xu, et al., "Activating cryptic biosynthetic gene cluster through a CRISPR-Cas12a-mediated direct cloning approach," *Nucleic Acids Research* (2022): 3581. <https://doi.org/10.1093/nar/gkac181>.

[6] H. L. Wang, Z. Li, R. N. Jia, et al., "RecET direct cloning and Red alpha beta recombineering of biosynthetic gene clusters, large operons or single genes for heterologous expression," *Nature Protocols* (2016): 1175. <https://doi.org/10.1038/nprot.2016.054>.

[7] W. Wang, H. He, H. Liu, et al., "Developing a robust genome editing tool based on an endogenous type I-B CRISPR-Cas system in *Saccharopolyspora spinosa*," *Sci China Life Sci* (2025). <https://doi.org/10.1007/s11427-024-2869-x>.

[8] T. J. Pawar, S. X. Ramos-Cruz, I. Bonilla-Landa, et al., "Quantitative NMR for detection of spinosad residues in agricultural soils," *Rsc Advances* (2025): 5547. <https://doi.org/10.1039/d5ra00356c>.

[9] C. Xue, X. Zhang, Z. Yu, et al., "Up-regulated spinosad pathway coupling with the increased concentration of acetyl-CoA and malonyl-CoA contributed to the increase of spinosad in the presence of exogenous fatty acid," *Biochemical Engineering Journal* (2013): 47. <https://doi.org/10.1016/j.bej.2013.10.004>.

[10] Z. L. Boynton, G. N. Bennett, F. B. Rudolph, "Intracellular concentrations of coenzyme A and its derivatives from Clostridium acetobutylicum ATCC 824 and their roles in enzyme regulation," *Applied and Environmental Microbiology* (1994): 39. <https://doi.org/10.1128/aem.60.1.39-44.1994>.
